# Tau Activates Transposable Elements in Alzheimer’s Disease

**DOI:** 10.1016/j.celrep.2018.05.004

**Published:** 2018-06-05

**Authors:** Caiwei Guo, Hyun-Hwan Jeong, Yi-Chen Hsieh, Hans-Ulrich Klein, David A. Bennett, Philip L. De Jager, Zhandong Liu, Joshua M. Shulman

**Affiliations:** 1 Department of Neuroscience, Baylor College of Medicine, Houston, TX 77030, USA; 2 Jan and Dan Duncan Neurologic Research Institute, Texas Children’s Hospital, Houston, TX 77030, USA; 3 Department of Molecular and Human Genetics, Baylor College of Medicine, Houston, TX 77030, USA; 4 Center for Translational and Computational Neuroimmunology, Department of Neurology, Columbia University Medical Center, New York, NY 10032, USA; 5 Cell Circuits Program, Broad Institute, Cambridge, MA 02142, USA; 6 Rush Alzheimer’s Disease Center, Rush University Medical Center, Chicago, IL 60612, USA; 7 Department of Pediatrics, Baylor College of Medicine, Houston, TX 77030, USA; 8 Department of Neurology, Baylor College of Medicine, Houston, TX 77030, USA; 9 These authors contributed equally; 10 Lead Contact

## Abstract

Aging and neurodegenerative disease are characterized by genomic instability in neurons, including aberrant activation and mobilization of transposable elements (TEs). Integrating studies of human postmortem brain tissue and *Drosophila melanogaster* models, we investigate TE activation in association with Tau pathology in Alzheimer’s disease (AD). Leveraging RNA sequencing from 636 human brains, we discover differential expression for several retrotransposons in association with neurofibrillary tangle burden and highlight evidence for global TE transcriptional activation among the long interspersed nuclear element 1 and endogenous retrovirus clades. In addition, we detect Tau-associated, active chromatin signatures at multiple *HERV-Fc1* genomic loci. To determine whether Tau is sufficient to induce TE activation, we profile retrotransposons in *Drosophila* expressing human wild-type or mutant Tau throughout the brain. We discover heterogeneous response profiles, including both age- and genotype-dependent activation of TE expression by Tau. Our results implicate TE activation and associated genomic instability in Tau-mediated AD mechanisms.

## In Brief

Integrating studies of human postmortem brain tissue and *Drosophila melanogaster* models, Guo et al. show that Alzheimer’s disease Tau neurofibrillary tangle pathology activates transcription of transposable element loci. An altered retrotransposon transcriptional landscape and associated genomic instability are implicated in Tau-mediated neurodegenerative mechanisms.

## Graphical Abstract

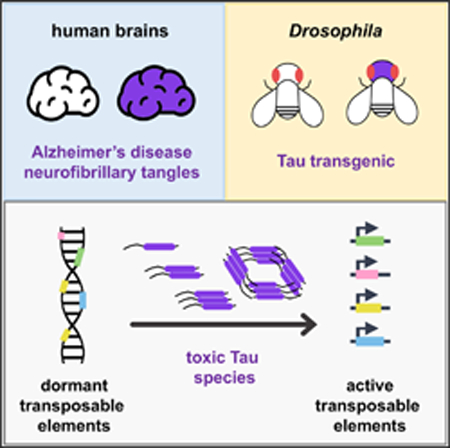

## INTRODUCTION

Alzheimer’s disease (AD) is the most common neurodegenerative disorder and the leading cause of dementia, with more than 13 million individuals projected to be affected in the United States by 2050 (Querfurth and LaFerla, 2010). At autopsy, AD is characterized by extracellular neuritic plaques and intracellular neurofibrillary tangles, comprised of aggregated, misfolded amyloid-β peptide and Tau protein, respectively. Tau pathology is also found in a heterogeneous group of neurodegenerative syndromes, the tauopathies, causing cognitive and/or motor impairment. Based on evidence from human postmortem material (Adamec et al., 1999) and animal models (Khurana et al., 2012), AD brain pathology is accompanied by genomic instability in affected neurons (Madabhushi et al., 2014), and Tau-mediated mechanisms are strongly implicated. In *Drosophila*, Tau induces global nuclear chromatin relaxation (Frost et al., 2014), abnormal transcriptional activation of heterochromatic genes, and DNA double-strand breaks (Khurana et al., 2012). Importantly, genetic manipulation of chromatin-modifying or DNA-repair pathways can suppress Tau neurotoxicity, suggesting that the maintenance of genomic integrity and neurodegeneration in AD may be causally linked rather than simply a downstream consequence of cell death.

Transposable elements (TEs) are mobile genetic sequences present in all eukaryotic genomes examined to date (Levin and Moran, 2011). Although TE-derived sequences are estimated to account for ~45% of the human genome, the majority are degenerate and incapable of mobilization. However, somatic transposition of TEs–specifically retrotransposons, which mobilize through an RNA intermediate–has been documented in adult neurons, including in human brains (Baillie et al., 2011; Evrony et al., 2012; Upton et al., 2015) and both mouse (Muotri et al., 2005) and fly models (Perrat et al., 2013). TE activation may be harmful, potentially disrupting the transcriptional landscape and triggering an immunologic response (Kassiotis and Stoye, 2016). With TE mobilization, somatic insertional mutagenesis and genomic rearrangements may also occur. Numerous systems have therefore evolved to suppress TE activity, and these mechanisms overlap with those regulating chromatin structure and DNA repair (Levin and Moran, 2011). However, TE surveillance may deteriorate with brain aging, leading to retrotransposon activation (Li et al., 2013; Maxwell et al., 2011; Wood et al., 2016). Based on studies in humans and animal models, aberrant TE activation has been implicated in many neurologic disorders, including multiple sclerosis (Morandi et al., 2017), Rett syndrome (Muotri et al., 2010), amyotrophic lateral sclerosis (ALS)-frontotemporal degeneration (FTD) (Douville et al., 2011; Li et al., 2015; Prudencio et al., 2017), and ataxia telangiectasia (Coufal et al., 2011). Evidence strongly suggests TEs may directly promote neuronal dysfunction and/or loss (Krug et al., 2017; Tan et al., 2012). For example, the RNA-binding protein, TDP-43, which aggregates in FTD-ALS, regulates the expression of TE transcripts (Li et al., 2012; Saldi et al., 2014), and inhibition of TE activation attenuates TDP-43 toxicity in fly models (Krug et al., 2017). Moreover, expression of the endogenous retrovirus (*HERV-K*) has been demonstrated in human cortical and spinal neurons in ALS, and the encoded Envelope (Env) protein is neurotoxic (Li et al., 2015).

Despite the evidence for genomic instability, retrotransposon expression has not been systematically evaluated in AD. In one small study, no differences in L1 genomic copy number were detected based on targeted PCR (Protasova et al., 2017). Here, we couple analyses of more than 600 human cortical transcriptomes with experiments in *Drosophila* transgenic models, highlighting global TE activation in AD and implicating Tau-mediated mechanisms.

## RESULTS

### Tau Pathologic Burden Is Associated with Altered TE Expression in Human Brains

**Figure 1.**
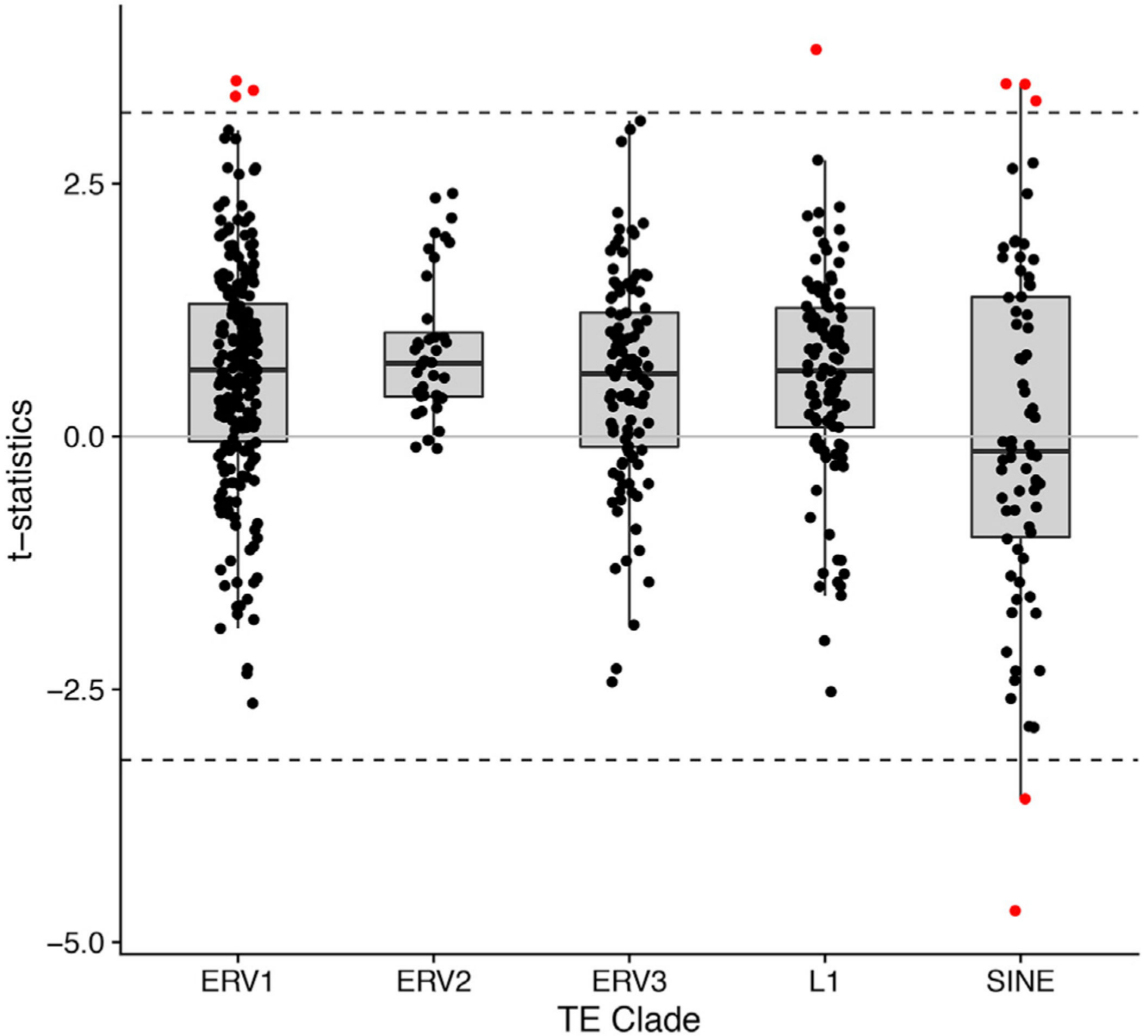
Tau Pathologic Burden Is Associated with Increased TE Expression in Human Brains Boxplots display regression t-statistics aggregated based on TE clade annotations. The dotted lines indicate the significance threshold, denoting those TEs (red) with the most extreme associations listed in Table 1. The mean t-statistic was significantly inflated for L1 (p = 7.1 × 10^−8^), ERV1 (p = 6.9 × 10^−14^), ERV2 (p = 1.9 × 10^−9^), and ERV3 (p = 8.2 × 10^−8^), consistent with a global impact of Tau pathologic burden on TE expression. See also Table S2.

To examine whether AD neurofibrillary tangle pathology is associated with TE activation, we first leveraged data from 2 prospective human clinical-pathologic studies, the Religious Orders Study and Rush Memory and Aging Project (ROSMAP). Our analyses included 636 deceased subjects with completed brain autopsies along with transcriptomic profiling of the dorsolateral prefrontal cortex based on RNA sequencing (RNA-seq). Clinical and demographic characteristics of our study cohort are detailed in Table S1. Current algorithms for building transcriptomes rely on alignment of RNA-seq data to a genomic reference, in which most repetitive sequences derived from TEs are excluded. In order to derive genome-wide estimates of transcription at TE loci in a computational efficient manner, we developed a tool, SalmonTE (Jeong et al., 2018). Based on a consensus TE sequence library from Repbase (Bao et al., 2015), we estimated transcriptional signatures for 366 long terminal repeat (LTR) and 181 non-LTR retrotransposons (Figure S1). We next applied linear regression, relating TE count estimates to a quantitative measure of average tangle burden, based on histologic counts from brain tissue sections. Table 1 highlights the 9 TEs significantly associated with Tau pathologic burden. Most TEs showed increased transcriptional activation, including selected long interspersed nuclear element 1 (LINE1 or L1), short interspersed nuclear elements (SINEs), and endogenous retroviruses (ERVs). To address specificity, we next examined each of the top-ranked TEs for associations with neuritic amyloid plaque pathology (Tables 1 and S3). Only a subset of the TE expression signatures was also associated with neuritic plaques, and the significance was attenuated. Given the small effect sizes and the large number of TEs, statistical power may be limited to detect associations for discrete TE expression signatures. We therefore performed a complementary analysis in which retrotransposons were aggregated based on clade membership, and the within-group distributions of t-statistic values for the association of TEs with tangles were evaluated. Interestingly, the ERV1, 2, 3, and L1 retrotransposon clades showed significant, positive deviation from the null distribution, consistent with global activation in the context of neurofibrillary tangle pathology (Figure 1; Table S2). Consistent TE clade activation patterns were also associated with AD pathologic diagnosis, and activation of the three ERV clades was related to global cognitive performance in the year proximate to death (Table S2). Our results suggest that the activity of TE loci may be broadly impacted by AD Tau pathology in human brains.

As introduced above, Tau pathology is associated with global chromatin reorganization and dysregulated gene expression (Frost et al., 2014; Klein et al., 2018). We hypothesized that Tau-induced chromatin relaxation might also de-repress silenced TEs. Among those elements implicated (Table 1), we focused on *HERV-Fc1*, which is unique for being present at low genomic copy number (n = 19 sites based on Dfam; Hubley et al., 2016). We leveraged an available chromatin immunoprecipitation sequencing (ChIP-seq) dataset, including 675 ROSMAP cortical samples, and extracted reads mapped to each *HERV-Fc1* genomic locus. Regression was performed to evaluate associations between tangle burden and level of histone 3, lysine 9 acetylation (H3K9Ac). Indeed, positive associations (p values < 0.05), indicating enhanced H3K9Ac, were detected at 4 out of 13 regions with available data; 3 regions remained significant after adjustment for multiple testing (Table S4). Our results support a hypothetical causal chain, in which Tau pathology promotes chromatin relaxation and TE transcriptional activation.

### Tau Is Sufficient to Alter TE Activity in the *Drosophila* Adult Nervous System

**Figure 2.**
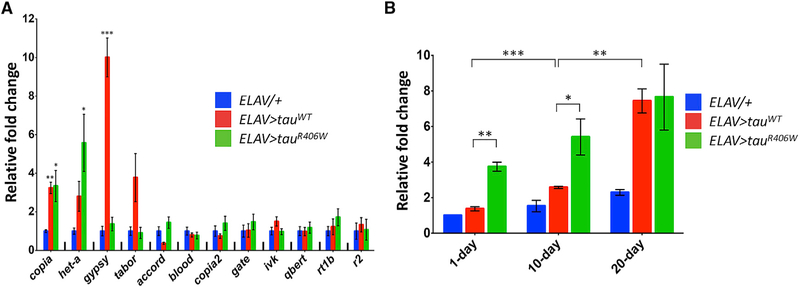
Tau Activates Expression of Selected TEs in the *Drosophila Brain* (A) In 20-day-old animals, the *copia*, *het-a*, and *gypsy* retrotransposons were activated following neuronal expression of wild-type and/or mutant human Tau. Expression of 12 TEs was profiled by qPCR in fly heads from the following genotypes: (1) *ELAV-GAL4/+*; (2) *ELAV-GAL4/+; UAS-Tau*^*WT*^*/+*; and (3) *ELAV-GAL4/+;UAS-Tau*^*R406W*^*/+*. One-way ANOVA model F-test was significant (p < 0.05) for *copia*, *het-a*, and *gypsy*. Analyses of 1- and 10-day-old animals are shown in Figure S2. (B) Expression of the *copia* retrotransposon is enhanced by age and mutant Tau. Two-way ANOVA testing was significant (p < 0.0001) for both age and genotype. All results (A and B) were normalized to *RpL32* expression, and fold-change relative to 1-day-old *ELAV-GAL4/+* control flies is shown (mean ± SEM). Subsetted t tests were performed for post hoc comparisons. *p < 0.05; **p < 0.01; ***p < 0.001.

In order to determine whether Tau pathology can induce activity of TE genomic loci, we turned to an established fly model relevant to AD (Wittmann et al., 2001). Pan-neuronal expression of either the wild-type human *MAPT* gene (*Tau*^*WT*^) or a mutant form associated with familial FTD (*Tau*^*R406W*^) causes age-dependent neuronal loss in association with hyperphosphorylated, misfolded Tau protein in the adult fly brain. We initially profiled 12 *Drosophila* TEs, including 8 LTR retrotransposons and 4 non-LTR retrotransposons, which have been previously demonstrated to be active in the fly nervous system, either following aging and/or manipulation of TE surveillance mechanisms (Li et al., 2013; Perrat et al., 2013). *Tau*^*WT*^, *Tau*^*R406W*^, or control flies were aged, and qRT-PCR was performed to assess TE transcriptional activity in adult heads. Three out of 12 TEs were significantly increased in one or both of the Tau transgenic lines at 20 days (Figure 2A), including both LTR (*copia* and *gypsy*) and L1-like, non-LTR retrotransposons (*het-a*). Notably, the *gypsy* TE is an endogenous insect retrovirus (Marlor et al., 1986) similar to human ERVs, including *HERV-Fc1*. The *Drosophila* retroelements were each increased ~3- to 10-fold in Tau transgenic flies compared with age-matched controls. Tau-dependent TE activation was already apparent in 1-day-old animals (Figure S2A), and for *copia*, expression increased progressively with aging (Figure 2B). Whereas *copia* and *het-a* showed evidence of enhanced activation in *Tau*^*R406W*^ animals, consistent with the increased toxicity of mutant Tau, expression of the *gypsy* TE was selectively increased in *Tau*^*WT*^ animals. Several other TEs were either unaffected or showed modest Tau-dependent reductions in expression when compared to age-matched control animals (Figures 2A and S2). Our results indicate that Tau is sufficient for activating expression of several *Drosophila* TEs in neurons but that the response profile is dependent on Tau genotype (wild-type versus mutant), aging, and the specific element examined. These findings were confirmed using an available RNA-seq dataset, permitting comprehensive assessments of TE expression signatures in 20-day-old *Tau*^*WT*^ versus control flies (Table S6). Our results reveal significant Tau-triggered expression changes affecting 64 out of 162 total retroelement signatures assayed (40%), including *gypsy*, *copia*, and *het-a* along with many other TEs. Out of the 37 Gypsy-class TE differential expression signatures, 22 (59%) demonstrated positive changes consistent with Tau-dependent activation (mean: 2.0; range: 1.3-to 26.6-fold increase). As a further control, the *Tau*^*WT*^ and *Tau*^*R406W*^ strains were each independently backcrossed to *w*^*1118*^ controls for 5 generations, ensuring a homogeneous genetic background. qPCR confirmed Tau-dependent increases in *het-a*, *copia*, and *gypsy* expression in aged animals (Figure S2C). Interestingly, we also detected modest but significant Tau- and age-dependent TE copy number increases based on qPCR of genomic DNA prepared from adult fly heads (Figure S2D), potentially consistent with retrotransposition (see below).

## DISCUSSION

**Figure 3.**
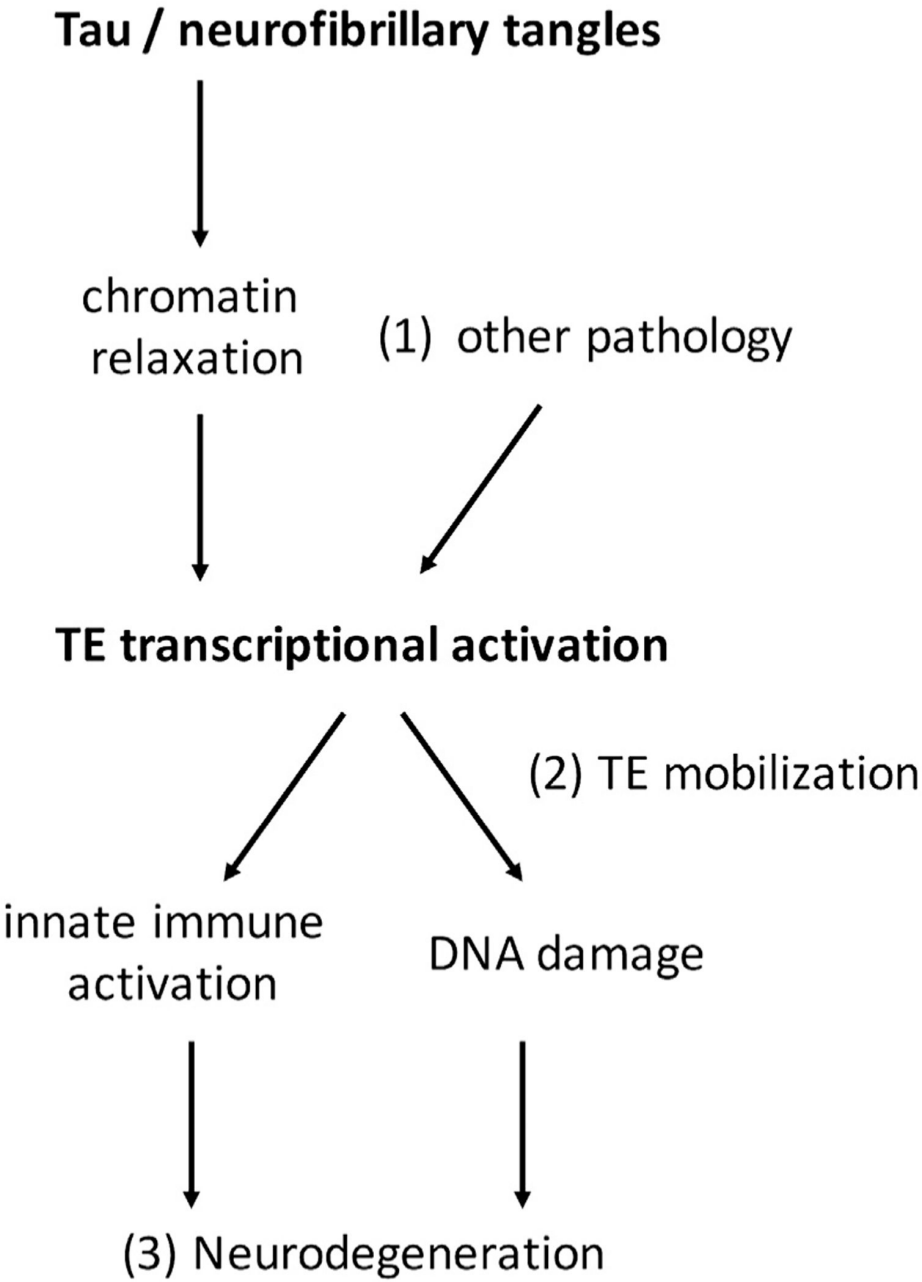
Hypothetical Model and Remaining Questions Our results along with other published evidence inform a causal model for Tau-mediated TE activation in AD, along with key knowledge gaps for further investigation (1–3). Tau is sufficient to induce TE transcriptional activation. Analyses of *HERV-Fc1* suggest that chromatin changes may in part be responsible, but other mechanisms may also contribute. (1) Besides Tau, it is likely that additional brain pathologies promote TE activation. (2) It remains to be determined whether TE transcriptional activation in AD leads to mobilization, potentially contributing to DNA damage and genomic instability. In the absence of transposition, TE expression may provoke an innate immune response. (3) Although DNA damage and neuroinflammation are strongly implicated in AD neurodegeneration, additional studies will be required to assess whether TEs contribute.

Our results, based on a cross-species strategy, implicate an altered TE transcriptional landscape in the setting of AD. Analyses of human brain transcriptomes identify differential retrotransposon expression signatures in association with neurofibrillary tangle burden along with evidence for widespread activation of selected TE clades, including L1 and the ERVs. L1 retrotransposons have previously been found to be activated in Rett syndrome (Muotri et al., 2010) and ataxia telangiectasia (Coufal et al., 2011), and ERV induction has been associated with ALS (Douville et al., 2011; Li et al., 2015) and multiple sclerosis (Morandi et al., 2017), including the same *HERV-Fc1* element detected in our analysis. We further discovered evidence of Tau-associated, active chromatin marks at genomic sites known to harbor *HERV-Fc1*. However, studies of human postmortem data in isolation are unable to establish causation. Moreover, Tau pathology co-exists in AD with neuritic amyloid plaques and other age-related brain lesions (Kapasi et al., 2017), making it difficult to establish specificity. We therefore turned to *Drosophila* transgenic models, revealing that Tau is sufficient to activate numerous TEs. For selected retrotransposons, activation was further enhanced with aging and by a mutant form of Tau associated with increased neurotoxicity. We propose a model (Figure 3) in which Tau modulates transcriptional activity at TE loci, possibly via chromatin remodeling, leading to neuronal dysfunction and/or loss. It is likely that other brain pathologies besides tangles also contribute to TE activation in AD. In our analyses, TE expression was also associated–albeit more weakly–with neuritic amyloid plaque pathologic burden. TDP-43 pathology, which commonly occurs in brains affected by AD (Kapasi et al., 2017), has also been associated with TE activation in human brains (Li et al., 2012, 2015) and fly models (Krug et al., 2017).

TE activation might both arise from and further promote genomic instability in AD. In both human brains and animal models, AD pathologic changes have been associated with epigenomic remodeling and transcriptional dysregulation (Frost et al., 2014; Gjoneska et al., 2015; De Jager et al., 2014). In ROSMAP, Tau pathology was related to widespread alterations in histone acetylation and similar changes were not associated with amyloid-β pathology (Klein et al., 2018). Expression of human Tau in the *Drosophila* brain also causes global chromatin relaxation and aberrant transcriptional activation of many genes that are usually repressed in heterochromatin (Frost et al., 2014). This suggests a potential mechanism for TE activation, because maintenance of these loci in a transcriptionally silent, heterochromatic state is one important mechanism for TE suppression (Levin and Moran, 2011). Based on public databases, the TEs most strongly implicated in our analyses (Table 1) are highly duplicated in the human genome. Moreover, our RNA-seq-based alignment strategy does not permit definitive localization of the genomic site(s) of origin for TE transcriptional activation. For example, the top-ranked *AluYh9* SINE element maps to 392 distinct genomic positions. For *HERV-Fc1*, which is present at comparatively low copy number, we provide additional evidence in support of our model, highlighting H3K9Ac chromatin marks at several loci consistent with active and/or relaxed chromatin conformation and resulting transcriptional activation. Importantly, genetic manipulations that restore chromatin packing in *Drosophila* have been demonstrated to rescue neuronal loss (Frost et al., 2014), indicating that these changes are causally linked to Tau-mediated neurodegeneration.

Aberrant TE expression may be highly damaging to neurons (Figure 3). Beyond the potential for TE mobilization (see below), with associated genomic rearrangements and insertional muta-genesis, even isolated transcriptional activation could be harmful. Both the innate and adaptive arms of the immune system can recognize retrotransposon-derived transcripts and/or proteins, provoking potent neuroinflammatory responses (Kassiotis and Stoye, 2016). Because the majority of TEs in the human genome have internal deletions or mutations that render them incompetent for autonomous mobilization, aberrant expression and activation of endogenous immunologic surveillance may be the most important implication of our findings. Indeed, TE-triggered immune reactions have been suggested in both multiple sclerosis (Antony et al., 2004) and ALS (Douville et al., 2011). In age-related macular degeneration, the accumulation of SINE *Alu* RNAs has been linked to an innate immune response that causes degeneration of the retinal pigment epithelium and resulting blindness (Tarallo et al., 2012). Neuroinflammation has also been implicated in AD pathogenesis, where the inciting events remain incompletely defined (Heppner et al., 2015).

One limitation of our study design is that we are unable to directly address whether retrotransposons mobilize causing new genomic insertions. In *Drosophila*, TEs can mobilize in the adult brain (Perrat et al., 2013), and *de novo* L1 insertions have been similarly documented in human brains and neural progenitor cells (Baillie et al., 2011; Coufal et al., 2009; Evrony et al., 2012; Upton et al., 2015). Our analyses suggest global activation of L1 elements in AD (Figure 1). However, of the 9 discrete TEs showing significant Tau-associated expression changes (Table 1), none are predicted to be capable of autonomous transposition. For example, based on Repbase, *L1MB4_5* is a “degenerate” L1 element lacking an intact open reading frame 2, which encodes a protein (ORF2p) with reverse transcriptase and endonuclease activity. Notably, most L1 retrotransposition in the human genome is believed to be due to the evolutionary young L1H subfamily, and such elements were absent from our top hits (Brouha et al., 2003). We also identify Tau-associated changes in the expression of several SINE retrotransposons. Although not capable of autonomous replication, SINE transposition can be facilitated by ORF2p provided by an active L1 element. Of the implicated SINEs, *AluYc5* is an evolutionary young Alu element, which is among the most abundant and active TEs in the human genome (Deininger, 2011). *HERV-Fc1* is an LTR retrotransposon including a *gag*, *pol*, and *env* gene flanked by two LTRs, but only the *env* open reading frame is known to be intact (Bénit et al., 2003). Selective expression of the Env protein from the related *HERV-K* is neurotoxic in human neuronal cultures and mouse models (Li et al., 2015), consistent with a similar model in AD, in which TE expression, but not mobi lization, may be sufficient for neuronal injury. Our finding of Tau-induced increases in genomic copy number for the *copia*, *gypsy*, and *het-a* TEs in flies (Figure S2) is potentially compatible with retrotransposition; however, this evidence is indirect and should be interpreted cautiously because alternative explanations are possible (e.g., amplification of existing genomic loci). Definitive evidence of Tau-induced TE transposition will require genomic sequencing to identify potential new insertion sites.

Prior investigations in both animal models and human postmortem tissue have documented DNA double-strand breaks and activation of repair pathways in association with AD pathologic changes (Madabhushi et al., 2014). Whereas numerous triggers have been proposed, including oxidative injury, neuronal excitability, and chromatin disruption, TE activation and/or mobilization may also contribute to genomic instability in AD (Figure 3). There is significant overlap between regulators of the DNA damage response and TE surveillance mechanisms (Levin and Moran, 2011). Importantly, manipulation of many such factors, including *loki* (homolog of human *CHEK2*), *atm*, *p53*, and *Ago3*, are potent modulators of Tau-induced neurodegenerative phenotypes in *Drosophila* (Khurana et al., 2012; Frost et al., 2014). In future work, it will be essential to determine whether TEs are causally linked to DNA damage and genomic instability in AD and whether this contributes to neurodegeneration.

## EXPERIMENTAL PROCEDURES

For detailed methods, see Supplemental Experimental Procedures.

### Analysis of TE Expression from Human Brain Transcriptomes

ROSMAP participants were free of known dementia at enrollment, agreed to annual clinical evaluations, and signed an informed consent and Anatomic Gift Act donating their brains at death, approved by the Institutional Review Board at Rush University. The modified Bielschowsky silver stain was used to visualize and quantify neurofibrillary tangles and neuritic plaques. ROSMAP RNA-seq data were generated with Illumina Hi-Seq. TE expression signatures were estimated using SalmonTE (Jeong et al., 2018) and a reference library derived from Repbase (Bao et al., 2015; Figure S1A). In selected cases, TE expression was independently confirmed by PCR (Figure S1B). Linear regression was performed to examine the relation of each TE expression signature (batch-corrected, log-transformed transcripts per million) with the neurofibrillary tangle burden outcome, adjusting for age at death, postmortem interval (PMI), and RNA integrity number. Statistical significance was based on a Benjamini-Hochberg false discovery rate (FDR) < 0.1. In order to evaluate associations for each TE clade, t-statistic values were aggregated from our primary regression model (Table S5), and a one-sample t test was performed to evaluate for a non-zero mean t-statistic value (Figure 1; Table S2). For the analyses of chromatin remodeling at *HERV-Fc1* loci, a log-linear regression model was implemented to evaluate associations between neurofibrillary tangle burden and H3K9Ac ChIP-seq reads, adjusting for library size, batch, PMI, cross-correlation, age, and gender.

### Analysis of TE Expression in *Drosophila* Heads

The *UAS-Tau*^*WT*^ and *UAS-Tau*^*R406W*^ transgenic flies were previously described (Wittmann et al., 2001). The following genotypes were used: (1) *ELAV-GAL4/+* (control); (2) *ELAV-GAL4/+; UAS-Tau*^*WT*^*/+*; and (3) *ELAV-GAL4/+; UASTau*^*R406W*^*/+*. qPCR was performed from total RNA prepared from fly heads in triplicate samples, using *RpL32* as an internal control. Expression (ΔΔC_T_ values) was normalized to that of 1-day-old control animals. One-way ANOVA was performed to detect differences between group mean expression values, considering each TE and time point separately. Subsetted t tests (two-tailed) were subsequently performed for post hoc comparisons of each Tau genotype with control animals. For *copia*, two-way ANOVA was secondarily performed to differentiate genotype and age effects. Error bars in all analyses represent the SEM. RNA-seq data from *ELAV-GAL4/+; UAS-Tau*^*WT*^*/+* or control (*ELAV-GAL4/+*) fly heads were analyzed using SalmonTE. Statistical significance was set to FDR < 0.1. For analyses of TE copy number, genomic DNA was extracted from heads of 10-day-old flies prior to qPCR.

## Supplementary Material

1

2

3

4

## Figures and Tables

**Table 1.** TEs Significantly Associated with Human Brain Tau Pathologic Burden

			Tangles		Plaques	
TE	Class	Clade	β	p Value	β	p Value
*AluYh9*	non-LTR	SINE	−0.033	3.34 × 10^−6^	−0.023	1.10 × 10^−5^
*L1MB4_5*	non-LTR	L1	0.032	1.43 × 10^−4^	0.008	0.20
*AluSp*	non-LTR	SINE	−0.024	3.66 × 10^−4^	−0.006	0.22
*HERV-Fc1*	LTR	ERV1	0.027	4.72 × 10^−4^	0.011	0.05
*AluYc5*	non-LTR	SINE	0.033	5.17 × 10^−4^	0.007	0.33
*THER2*	non-LTR	SINE	0.053	5.30 × 10^−4^	0.021	0.08
*PRIMA4_LTR*	LTR	ERV1	0.025	6.65 × 10^−4^	0.016	4.82 × 10^−3^
*LTR77*	LTR	ERV1	0.026	8.14 × 10^−4^	0.007	0.26
*PB1D11*	non-LTR	SINE	0.022	9.51 × 10^−4^	0.013	7.39 × 10^−3^

Top-ranked associations with tangle pathology are shown (false discovery rate < 0.1); comprehensive results for 547 retrotransposons are shown in Table S5. Only a subset of TEs was associated with amyloid plaques. β, beta coefficient.

## Highlights

- Alzheimer’s Tau pathology is related to retrotransposon expression in human brains
- Neurofibrillary tangles also associate with chromatin relaxation at selected loci
- Tau activates transposable element transcription in transgenic flies
