# Supplementary material for: Tau Activates Transposable Elements in Alzheimer’s Disease
Source: Cell Rep. Author manuscript; Available in PMC 2018 Oct 11. (PMC6181645; doi:10.1016/j.celrep.2018.05.004)
Supplement: 1 [file NIHMS1506631-supplement-1.pdf]

Cell Reports, Volume *23*

Supplemental Information

Tau Activates Transposable Elements in Alzheimer's Disease

Caiwei Guo, Hyun-Hwan Jeong, Yi-Chen Hsieh, Hans-Ulrich Klein, David A. Bennett, Philip L. De Jager, Zhandong Liu, and Joshua M. Shulman

SUPPLEMENTAL FIGURES

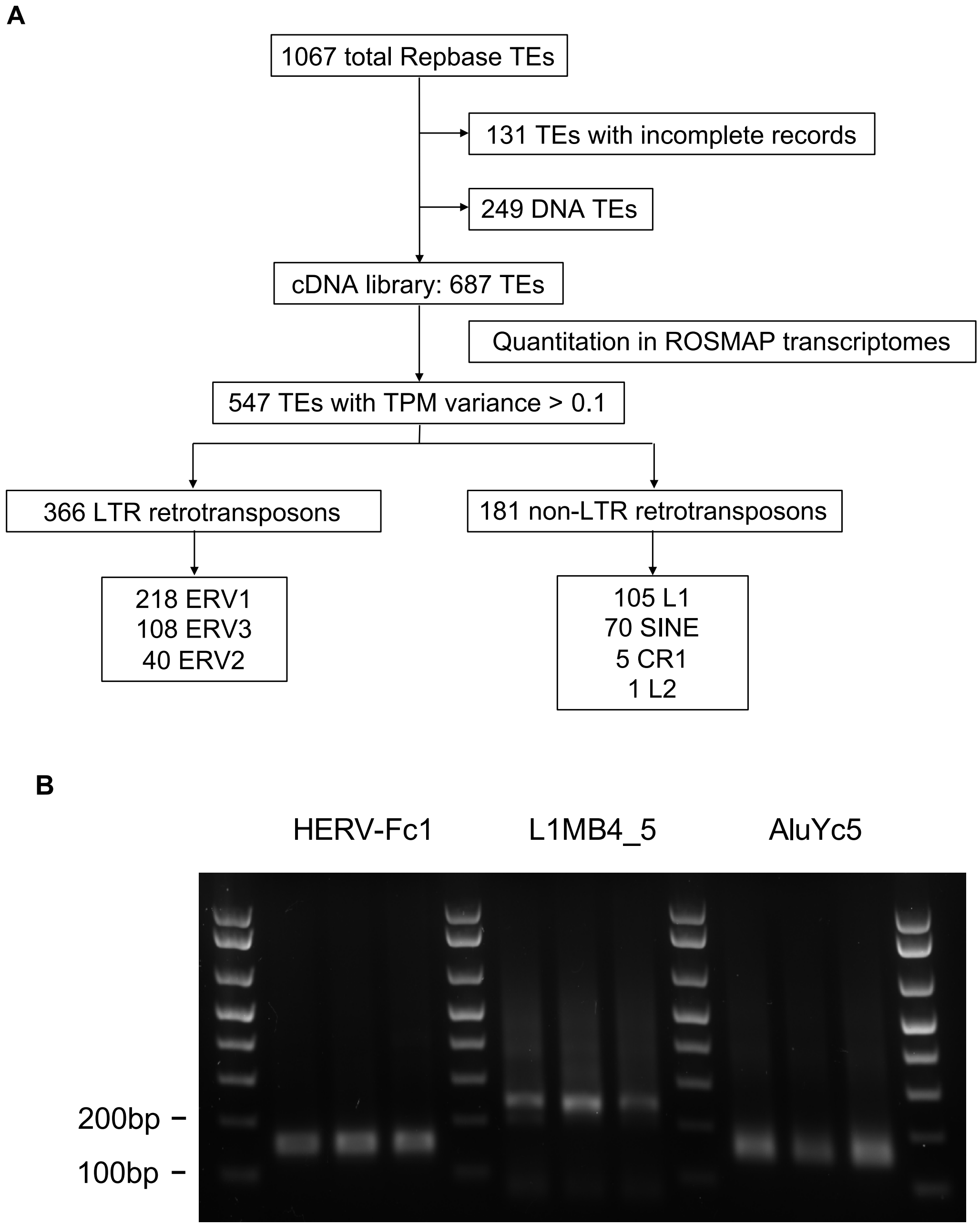

Figure S1. Profiling of TEs in human brain transcriptomes (Related to Figure 1).
(A) Schematic overview of the establishment of a human TE library for use in this study. (B) PCR validation of selected human TEs in 3 independent cortical RNA samples from ROSMAP postmortem brain tissue specimens. Agarose gel electrophoresis of PCR products for HERV-Fc1 (155bp), L1MB4_5 (219bp), and AluYc5 (142bp) are shown.

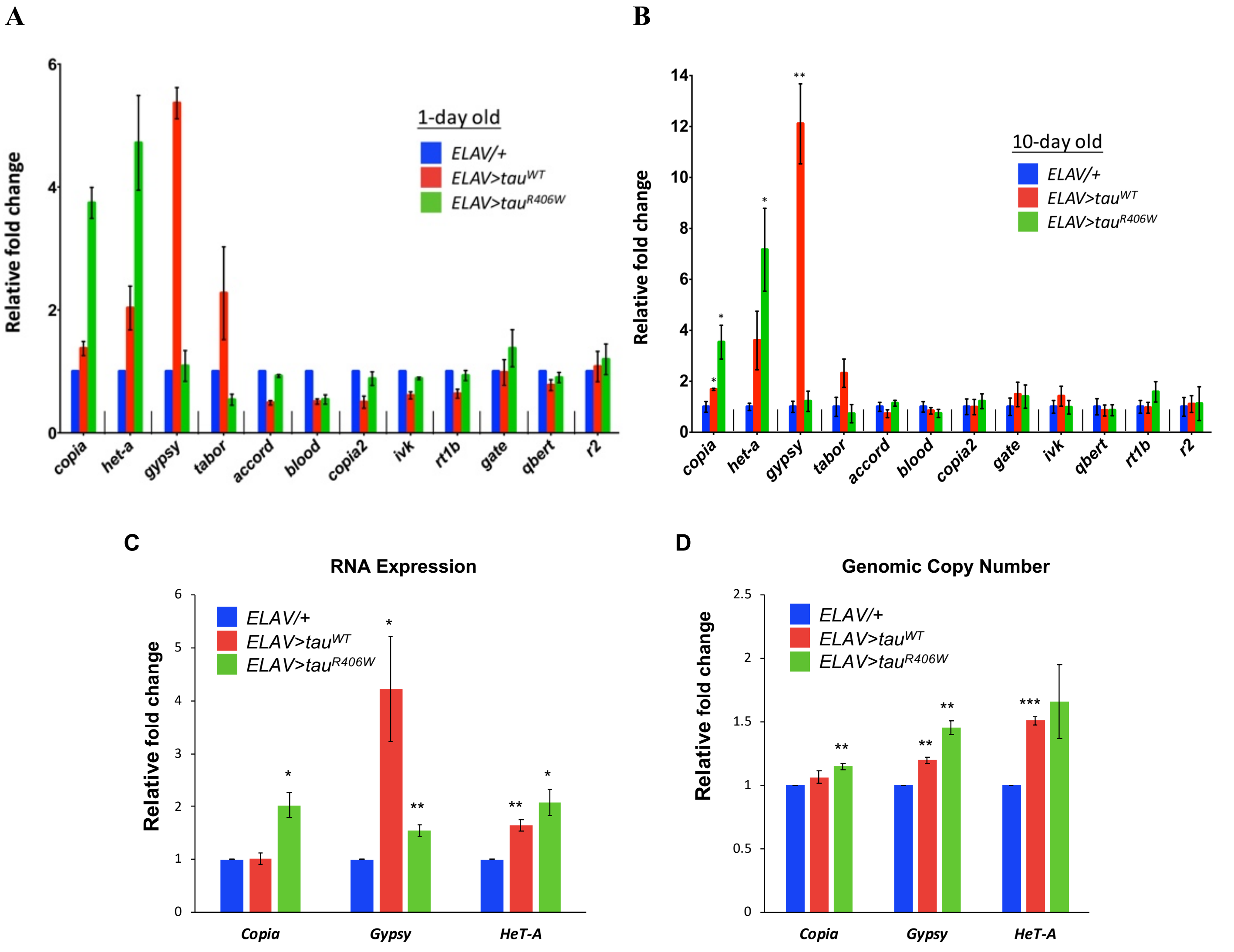

Figure S2. Tau activates TEs in the *Drosophila* brain (Related to Figure 2).
(A, B) Expression of 12 TEs was profiled by qPCR in triplicate samples from heads of *ELAV-GAL4/+*, $ELAV>Tau^{WT}$, and $ELAV>Tau^{R406W}$ in 1-day-old (A) and 10-day-old (B) animals. Analyses conducted in 20-day-old animals are shown in Figure 2. (C, D) As a further control, the $UAS\text{-}Tau^{WT}$ and $UAS\text{-}Tau^{R406W}$ strains were back-crossed to w^{1118} control flies for 5 generations, ensuring a homogenous genetic background among our control and experimental genotypes. mRNA expression (C) or genomic copy number (D) of *copia*, *gypsy*, and *het-a* was profiled by qPCR in the heads of *ELAV-GAL4/+*, $ELAV>Tau^{WT}$, and $ELAV>Tau^{R406W}$ in 10-day-old animals which were back crossed to w^{1118} flies for 5 generations. Since the 1-day old *ELAV-GAL4/+* control data was used for normalization, statistical analysis was not performed for comparisons at 1-day (A). For all other experiments (B-D), one-way ANOVA was performed to detect differences between group means, considering each TE separately (model F-test $p<0.05$ for *copia*, *het-a*, *gypsy*). Subsetted t-tests were subsequently performed for post-hoc comparisons of each Tau genotype with control animals. All results were normalized to *RpL32* and fold-change relative to *ELAV/+* control flies is shown (mean±S.E.M.). *, $p<0.05$, **, $p<0.01$, ***, $p<0.001$.

Table S1. ROSMAP autopsy cohort clinical and demographic characteristics (Related to Table 1).

**n**	636
**Age at enrollment, mean yr (SD)**	81.3 (7.0)
**Age at death, mean yr (SD)**	88.7 (6.7)
**Male, n**	229 (36.0%)
**Education, mean yr (SD)**	16.4 (3.5)
**No Cognitive Impairment, n**	201 (31.7%)
**Mild Cognitive Impairment, n**	168 (26.5%)
**Clinical AD, n**	254 (40.0%)
**Postmortem interval, mean hrs (SD)**	7.3 (4.8)
**Pathologic AD, n**	385 (60.5%)
**Neurofibrillary Tangle Burden, mean (SD)**	0.6 (0.8)
**Braak Score, n**	
**0**	7 (1.1%)
**I**	51 (8.0%)
**II**	54 (8.5%)
**III**	175 (27.5%)
**IV**	210 (33.0%)
**V**	132 (20.8%)
**VI**	7 (1.1%)

Clinical and pathologic diagnoses of AD were based on NINCDS-ADRDA and NIA-Reagan criteria, respectively. Neurofibrillary tangle pathology based on averaged pathologic counts from silver-stained tissue sections.

Table S2. TE clade associations with tangles, AD pathology, and cognition (Related to Figure 1).

		tangles		AD diagnosis		cognition	
**TE clade**	**n**	**mean (SE)**	**p-value**	**mean (SE)**	**p-value**	**mean (SE)**	**p-value**
ERV1	218	0.61 (0.08)	6.94×10^{-14}	0.50 (0.06)	1.45×10^{-14}	0.46 (0.07)	6.56×10^{-10}
ERV2	40	0.87 (0.11)	1.91×10^{-9}	0.62 (0.10)	2.35×10^{-7}	0.44 (0.12)	7.00×10^{-4}
ERV3	108	0.57 (0.10)	8.16×10^{-8}	0.47 (0.09)	5.12×10^{-7}	0.40 (0.11)	3.21×10^{-4}
L1	105	0.59 (0.10)	7.08×10^{-8}	0.28 (0.09)	1.18×10^{-3}	0.09 (0.09)	0.34
SINE	70	0.04 (0.21)	0.86	-0.02 (0.15)	0.97	-0.10 (0.19)	0.95

To evaluate summary associations for each TE clade, t-statistics were extracted from the regression model, and aggregated based on Repbase classifications. A one-sample t-test (two-tailed) was performed to evaluate for a non-zero mean t-statistic for each clade. Secondary analyses additionally examined associations between each TE clade and either consensus AD pathologic diagnosis (Bennett et al., 2006; National Institute on Aging, 1997) or global cognitive performance (Wilson et al., 2015), using logistic or linear regression, respectively. All analyses were adjusted for age at death, PMI and RIN, and the analysis for cognitive performance was additionally adjusted for sex and years of education. The mean t-statistic, standard error (SE), and p-value is shown for each TE clade. The results for neurofibrillary tangles correspond to the data presented in Figure 1.

Table S3. Alternate regression models to assess contributions of plaques and tangles (Related to Table 1).

TE	R-Squared (p-value)		
	**Model 1**	**Model 2**	**Model 3**
AluYh9	0.079 ($1.4x10^{-10}$)	0.076 ($4.31x10^{-10}$)	0.086 ($5.27 x10^{-11}$)
L1MB4_5	0.042 ($1.8x10^{-5}$)	0.023 ($6.05x10^{-3}$)	0.044 ($3.39 x10^{-5}$)
AluSp	0.060 ($6.4x10^{-8}$)	0.043 ($1.29x10^{-5}$)	0.061 ($1.55 x10^{-7}$)
HERV-Fc1	0.030 ($6.3x10^{-4}$)	0.017 (0.03)	0.030 ($1.56 x10^{-3}$)
AluYc5	0.025 ($3.2x10^{-3}$)	0.008 (0.31)	0.027 ($4.39 x10^{-3}$)
THER2	0.023 ($5.8x10^{-3}$)	0.009 (0.23)	0.023 (0.01)
PRIMA4_LTR	0.051 ($1.1x10^{-6}$)	0.046 ($5.94x10^{-6}$)	0.053 ($1.85 x10^{-6}$)
LTR77	0.028 ($1.3x10^{-3}$)	0.013 (0.09)	0.029 ($2.31 x10^{-3}$)
PB1D11	0.028 ($1.5x10^{-3}$)	0.021 ($7.78x10^{-3}$)	0.029 ($2.19 x10^{-3}$)

Alternative regression models were considered relating TE expression with AD pathology. All models were similarly adjusted for age, postmortem interval (PMI), and RNA integrity number (RIN). First, we examined the primary model (Model 1: TE ~ NFT + age + PMI + RIN) relating neurofibrillary tangle (NFT) pathology with TE expression signatures, noting the regression model R-squared and overall model F-test p-value. We next examined a similar adjusted model (Model 2: TE ~ NP + age + PMI + RIN), but considering the association with neuritic amyloid plaques (NP). Lastly, we considered a joint model (Model 3: TE ~ NFT + NP + age + PMI + RIN) including terms for both neuritic plaques and neurofibrillary tangles. The results reveal that including the neuritic plaque term explains little, if any, additional variance in TE expression beyond that already explained by neurofibrillary tangles. Only in 1 case (AluYh9) does the model p-value improve in the joint Model 3 compared Model 1.

Table S4. Tau-associated heterochromatin relaxation at *HERV-Fc1* loci (Related to Table 1).

Loci	Chr	StartPos	EndPos	Mean	SD	β	p-value
TE1	1	169685137	169688136	28.151	9.402	0.053	0.028
TE2	1	224886406	224889405	40.804	11.718	0.033	0.085
TE3	2	83874409	83879018	35.821	10.951	0.015	0.473
TE4	6	158884733	158887732	38.036	10.604	-0.012	0.502
TE5	7	64835238	64839812	167.942	40.821	0.006	0.579
TE6	7	153409350	153413807	19.483	6.507	0.036	0.142
TE7	11	5927657	5930656	20.893	7.200	-0.040	0.127
TE9	14	70297503	70300502	18.994	6.537	0.081	**0.001**
TE10	14	92046411	92049410	32.129	9.341	0.057	**0.004**
TE12	19	52754206	52757205	27.840	8.681	0.076	**0.001**
TE13	22	39059285	39062284	32.302	9.356	0.004	0.861
TE14	X	75967359	75970358	18.917	8.299	-0.006	0.832
TE15	Y	10050093	10053092	13.031	7.004	-0.007	0.823

HERV-Fc1 genomic loci were determined based on the Dfam database (Hubley et al., 2015). Coordinates are from GRCh37 (hg19) reference. A negative binomial regression model was fitted for each HERV-Fc1 locus to test the association between H3K9Ac levels and neurofibrillary tangle burden. Read counts from ChIP-seq were used as outcome and models were adjusted for library size, batch, post mortem interval, cross correlation, age and gender. Mean ChIP-seq read counts and standard deviation (SD) are shown for each locus evaluated, along with regression coefficient (β) and the unadjusted p-value. Following adjustment for multiple hypothesis testing (FDR<0.05), 3 loci remained significant (boldface).

SUPPLEMENTAL EXPERIMENTAL PROCEDURES

Human Autopsy Cohort

Religious Orders Study (ROS) and Rush Memory and Aging Project (MAP) participants were free of known dementia at enrollment, agreed to annual clinical evaluations, and signed an informed consent and Anatomic Gift Act donating their brains at death (Bennett et al., 2012a, 2012b). The ROS and MAP studies were approved by the Institutional Review Board at Rush University. Modified Bielschowsky silver stain was used to visualize neuritic plaques, diffuse plaques, and neurofibrillary tangles in tissue sections from the midfrontal, middle temporal, inferior parietal, and entorhinal cortices and the hippocampal CA1 sector (Bennett et al., 2006). As in prior work (Bennett et al., 2009), a quantitative composite score for neurofibrillary tangle or neuritic amyloid plaque pathologic burden was created by dividing the raw counts in each region by the population standard deviation of the region-specific counts and then averaging the scaled counts over the 5 brain regions to create standardized summary measures. Neuropathologic diagnosis of AD was made based on intermediate or high likelihood of AD by criteria from the National Institute on Aging and the Reagan Institute Working Group on Diagnostic Criteria for the Neuropathological Assessment of Alzheimer's Disease (National Institute on Aging, 1997). Level of cognition was based on 17 cognitive tests performed at annual clinical evaluations—the last evaluation within the year proximate to death was used. As in prior studies (Bennett et al., 2006), test results were converted to z-scores, using the mean and SD from the baseline evaluation of all participants, and averaged to yield a global summary measure of cognitive performance proximate to death. RNA-seq data was generated using postmortem brain tissue (dorsolateral prefrontal cortex) from ROS/MAP subjects, as previously described (De Jager et al., 2014). The RNA was extracted from the tissue after the quality control evaluation based on RIN (Schroeder et al., 2006) or RNA were extracted first, and then RIN was used to filter out low-quality samples. The library was sequenced using Illumina Hi-Seq with 101 bp reads and 4-plex pooling. 636 subjects had complete data including RNA-seq transcriptome profiles, neurofibrillary tangle burden outcome trait, and the pertinent covariates (Table S1). H3K9Ac chromatin-immunoprecipitation sequencing (ChIP-Seq) was also available from 675 ROSMAP brains (Klein et al., 2018; Ng et al., 2017).

Estimation and Analysis of TE Expression from Human Brain Transcriptomes

ROSMAP RNA-seq data were converted from binary sequence alignment map (BAM) format to FASTQ using Picard (https://broadinstitute.github.io/picard/). The consensus cDNA sequence library of human TEs was downloaded using the CENSOR webserver tool in Repbase (version 22.05, http://www.girinst.org/repbase/update/browse.php). As detailed in Figure S1A, our custom TE reference library included 687 retrotransposons for initial quantification from RNA-seq data, after excluding both simple repeats and multicopy genes, DNA transposons (which replicate without an RNA intermediate), and those elements lacking sufficient consensus sequence features to allow unambiguous classification in Repbase. We next used SalmonTE (version 0.6.0) (Jeong et al., 2018), which implements the Salmon algorithm (Patro et al. 2017) for efficient, alignment-free quantification of RNA-seq data to generate normalized TE counts (TPM, Transcripts Per Kilobase Million) for each ROSMAP brain transcriptome. In our prior published work (Jeong et al., 2018), the SalmonTE estimation algorithm yields comparable estimates to both qPCR and TETranscripts (Jin et al., 2015), a published tool for detection of TE expression from RNA-seq data. In selected cases, estimates of TE expression (HERV-Fc1, L1MB4, and AluYc5) in human brains were independently confirmed by PCR (Figure S1B), using the following primers:

HERV-Fc1	5'-TTGTCAGAGGCGAGGCAAAT-3'
	5'-TTAACGGCAAGAGAGGCTGG-3'
L1MB4	5'-AGACCACCTAAAGCCTGGGA-3'
	5'-GGCACTCCCCTTCAACACTT-3'
AluYc5	5'-GGAGATCGAGACCACGGTGA-3'
	5'-ACTGCAAGCTCCGCCTTCCG-3'

All TPM values were adjusted for potential RNA-seq batch effects using Combat (Johnson et al., 2007). Prior to batch correction, we excluded 140 TEs with low variability (TPM variance < 0.1), and the remaining count data for 547 TEs were quantile normalized and log2-transformed. The batch-corrected, log-transformed Transcripts Per Kilobase Million (TPM) outcome variable follows a Gaussian distribution and is suitable for linear regression. As in prior studies (Bennett et al., 2009), the neurofibrillary tangle trait was square-root transformed to approximate a normal distribution. We also included pertinent covariates to adjust for age at death, postmortem interval (PMI) and

RNA integrity (RIN). Thus, using the *lm* function in R our primary analysis tested the following statistical model: Combat(log(TPM)) ~ sqrt(NFT) + age of death + PMI + RIN. In order to account for multiple-comparisons, the Benjamini-Hochberg procedure was applied (Benjamini and Hochberg, 1995); statistical significance was based on a false discovery rate (FDR) < 0.1. Complete results of the association tests for 547 TEs with tangle pathologic burden is detailed in Table S5. The top-ranked TEs were secondarily evaluated for associations with the square-root transformed neuritic amyloid plaque trait (Table 1). In order to evaluate summary associations for each TE clade, t-statistic values were extracted from our primary regression model (Table S5), and aggregated for each clade, based on Repbase classifications. A one-sample t-test (two-tailed) was then performed to evaluate for a non-zero mean t-statistic value within each clade (Figure 1 & Table S2). Secondary analyses additionally examined associations between each TE clade and either consensus AD pathologic diagnosis (Bennett et al., 2006; National Institute on Aging, 1997) or global cognitive performance (Wilson et al., 2015), using logistic or linear regression, respectively. All analyses were adjusted for age at death, PMI and RIN, and the analysis for cognitive performance was additionally adjusted for sex and years of education.

For analysis of neurofibrillary tangle-associated chromatin changes, *HERV-Fc1* genomic loci were determined using the Dfam database and webserver (Hubley et al., 2015), based on the trusted and non-redundant filtering parameters. A 3kb genomic region was defined centered on each *HERV-Fc1* site, and we extracted reads mapped to these regions from the ChIP-seq data. Non-uniquely mapped reads and duplicated reads were removed. Regions with a mean of less than 10 reads across all samples were excluded. A log-linear regression model was implemented to evaluate associations between neurofibrillary tangle pathologic burden and level of H3K9Ac, and adjusting for library size, batch, pmi, cross-correlation, age and gender.

Drosophila Stocks and Husbandry

The *UAS-Tau*^{WT} and *UAS-Tau*^{R406W} transgenic flies were previously described (Wittmann et al., 2001). Pan-neuronal expression was achieved using the GAL4-UAS system (Brand and Perrimon, 1993), in which Tau cDNAs under the control of the yeast upstream activation sequence (UAS) are responsive to the *GAL4* transcriptional activator, directed to the nervous system via *ELAV* regulatory elements (*ELAV-GAL4*). Experimental animals consisted of the following genotypes: *ELAV-GAL4/+;UAS-Tau*^{WT}*/+* and *ELAV-GAL4/+;UAS-Tau*^{R406W}*/+*. *ELAV-GAL4/+* was used as a control. All flies were raised on standard *Drosophila* media at 25°C, and aged to 1-, 10-, or 20-days.

Analysis of TE Expression in _Drosophila_ Heads

Approximately 100 adult flies, equally divided between males and females, were collected for each genotype at the specified ages. Frozen fly heads were homogenized in Trizol (Invitrogen) and treated with DNaseI (Promega). Total RNA was extracted using the RNeasy Micro Kit (Qiagen). Following reverse transcription using the SuperScript III First-Strand Synthesis System (Invitrogen), quantitative reverse transcription PCR was performed using iQ SYBR Green Supermix (Biorad) in a CFX96 Touch Real-Time PCR Detection System (Biorad) with standard cycling parameters. Each reaction was performed in triplicate. *RpL32* was used as endogenous control for normalization of each transposon to calculate ΔC_T values. We used the following PCR primers:

accord (Chung et al., 2007)	5'-CGTGAGTTACGGGTGCCTCCG-3'
	5'-AGTTACCATGCCCAGCATTAAC-3'
copia (Kalmykova et al., 2005)	5'-GCATGAGAGGTTTGGCCATATAAGC-3'
	5'-GGCCCACAGACATCTGAGTGTACTACA-3'
gate (Klenov et al., 2011)	5'-CCTTTGGGAAGCAGGCGTAAA-3'
	5'-TCTTCAGACATAGGAGAGAGCGGC-3'
het-a	5'-CGGCGAACGAGTTCTGGAAT-3'
	5'- AGTTGTGTACTTGGGCTGGG-3'
r2	5'-TCCATTCTGCCACCCTGAAC-3'
	5'-GGAAAACCCGCTCAGGTACA-3'
rt1b	5'-TGGCCAACAGAGTGGATGTC-3'
	5'-ACTTCGGTGAGCAGGATGTG-3'
qbert	5'-TATACGGTCGCCTGTGAAGC-3'
	5'-GGCAGTGGTTGTGGGGATTT-3'
tabor	5'-GACTCTCTTAGACAGCGGCG-3'
	5'-ACGACTCCTTTACGACTGCC-3'
blood	5'-GAGGGGAGGTGTAGTATGTGC-3'

	5’-GCGTGGGGATGCTGACTTAG-3’
ivk	5’-TCAAGCCACCAACGAACGTA-3’
	5’-ATTGACTGCAGGCGTTGAGA-3’
copia2	5’-CGGATTTCGTGTGTCGTTCG-3’
	5’-CCGAATCTCTGGTGCCACTT-3’
gypsy (Liu et al., 2011)	5’-GTTCATACCCTTGGTAGTAGC-3’
	5’-CAACTTACGCATATGTGAGT-3’
RpL32 (Luce-Fedrow et al., 2008)	5’-ATCGGTTACGGATCGAACAA-3’
	5’-GACAATCTCCTTGCGCTTCT-3’

One-way ANOVA was performed to detect differences between group mean expression values, considering each TE and timepoint separately. Subsetted t-tests (two-tailed) were subsequently performed for post-hoc comparisons of each Tau genotype with control animals. For *copia*, a two-way ANOVA was secondarily used to differentiate genotype and age effects. Error bars in all analyses represent the standard error of the mean (SEM). In order to control for potential confounding due to genetic background, follow-up experiments were also performed to detect *gypsy, copia,* and *het-a* expression after the *UAS-Tau*^{WT} and *UAS-Tau*^{R406W} strains were back-crossed to *w*^{1118} flies for 5 generations (Figure S2C). For estimation of TE genomic copy number (Figure S2D), 20 frozen adult fly heads, equally divided between males and females, were collected for each genotype in triplicate. Genomic DNA was extracted using the PureLink Genomic DNA Mini Kit (ThermoFisher). Quantitative reverse transcription PCR was performed using the protocol and the primer sets for *copia*, *gypsy*, and *het-a* described above. *RpL32* was again used as an internal control.

For RNA-seq, total RNA was extracted from triplicate samples consisting of 100 frozen fly heads each from either 20-day-old *ELAV>Tau*^{WT} or controls (*ELAV-GAL4/+*), and sequencing was performed at the Broad Institute using Illumina HiSeq with 96bp paired-end reads. *Drosophila* TE annotations were downloaded from Repbase (version 22.05), and TE expression signatures were determined using SalmonTE, as above (Jeong et al., 2018). Statistical analysis was performed using DESeq2, implemented within SalmonTE, and using default parameters; significance was set to FDR<0.1. Comprehensive results of the *Drosophila* RNA-seq analysis are shown in Table S6.

SUPPLEMENTAL REFERENCES

Benjamini, Y., and Hochberg, Y. (1995). Controlling the false discovery rate: a practical and powerful approach to multiple testing. J. R. Stat. Soc. *57*, 289–300.

Bennett, D.A., Schneider, J.A., Arvanitakis, Z., Kelly, J.F., Aggarwal, N.T., Shah, R.C., and Wilson, R.S. (2006). Neuropathology of older persons without cognitive impairment from two community-based studies. Neurology *66*, 1837–1844.

Bennett, D.A., De Jager, P.L., Leurgans, S.E., and Schneider, J.A. (2009). Neuropathologic intermediate phenotypes enhance association to Alzheimer susceptibility alleles. Neurology *72*, 1495–1503.

Bennett, D.A., Schneider, J.A., Arvanitakis, Z., and Wilson, R.S. (2012a). Overview and findings from the religious orders study. Curr. Alzheimer Res. *9*, 628–645.

Bennett, D.A., Schneider, J.A., Buchman, A.S., Barnes, L.L., Boyle, P.A., and Wilson, R.S. (2012b). Overview and findings from the rush Memory and Aging Project. Curr. Alzheimer Res. *9*, 646–663.

Brand, A.H., and Perrimon, N. (1993). Targeted gene expression as a means of altering cell fates and generating dominant phenotypes. Development *118*, 401–415.

Chung, H., Bogwitz, M.R., McCart, C., Andrianopoulos, A., Ffrench-Constant, R.H., Batterham, P., and Daborn, P.J. (2007). Cis-regulatory elements in the accord retrotransposon result in tissue-specific expression of the Drosophila melanogaster insecticide resistance gene Cyp6g1. Genetics *175*, 1071–1077.

Hubley, R., Finn, R.D., Clements, J., Eddy, S.R., Jones, T.A., Bao, W., Smit, A.F.A., and Wheeler, T.J. (2015). The Dfam database of repetitive DNA families. Nucleic Acids Res. *44*, D81–D89.

De Jager, P.L., Srivastava, G., Lunnon, K., Burgess, J., Schalkwyk, L.C., Yu, L., Eaton, M.L., Keenan, B.T., Ernst, J., McCabe, C., et al. (2014). Alzheimer's disease: Early alterations in brain DNA methylation at ANK1, BIN1, RHBDF2 and other loci. Nat. Neurosci. *17*, 1156–1163.

Jeong, H., Yalamanchili, H.K., Guo, C., Shulman, J.M., and Liu, Z. (2018). An ultra-fast and scalable quantification pipeline for transposable elements from next generation sequencing data. Pac. Symp. Biocomput. *23*, 168–179.

Jin, Y., Tam, O.H., Paniagua, E., and Hammell, M. (2015). TEtranscripts: A package for including transposable elements in differential expression analysis of RNA-seq datasets. Bioinformatics *31*, 3593–3599.

Johnson, W., Li, C., and Rabinovic, A. (2007). Adjusting batch effects in microarray expression data using empirical Bayes methods. Biostatistics *8*, 118–127.

Kalmykova, A.I., Klenov, M.S., and Gvozdev, V.A. (2005). Argonaute protein PIWI controls mobilization of retrotransposons in the Drosophila male germline. Nucleic Acids Res. *33*, 2052–2059.

Klein, H.-U., McCabe, C., Gjoneska, E., Sullivan, S.E., Kaskow, B.J., Tang, A., Smith, R. V, Xu, J., Pfenning, A.R., Bernstein, B.E., et al. (2018). Epigenome-wide study uncovers tau pathology-driven changes of chromatin organization in the aging human brain. bioRxiv 273789. doi: https://doi.org/10.1101/273789

Klenov, M.S., Sokolova, O.A., Yakushev, E.Y., Stolyarenko, A.D., Mikhaleva, E.A., Lavrov, S.A., and Gvozdev, V.A. (2011). Separation of stem cell maintenance and transposon silencing functions of Piwi protein. Proc. Natl. Acad. Sci. *108*, 18760–18765.

Liu, L., Qi, H., Wang, J., and Lin, H. (2011). PAPI, a novel TUDOR-domain protein, complexes with AGO3, ME31B and TRAL in the nuage to silence transposition. Development *138*, 1863–1873.

Luce-Fedrow, A., Von Ohlen, T., Boyle, D., Ganta, R.R., and Chapes, S.K. (2008). Use of Drosophila S2 cells as a

model for studying Ehrlichia chaffeensis infections. Appl. Environ. Microbiol. *74*, 1886–1891.

National Institute on Aging, Reagan Institute Working Group on Diagnostic Criteria for the Neuropathological Assessment of Alzheimer's Disease. (1997). Consensus Recommendations For the Postmortem Diagnosis Of Alzheimers-Disease. Neurobiol. Aging *18*, S 1-S 2.

Ng, B., White, C.C., Klein, H.U., Sieberts, S.K., McCabe, C., Patrick, E., Xu, J., Yu, L., Gaiteri, C., Bennett, D.A., et al. (2017). An xQTL map integrates the genetic architecture of the human brain's transcriptome and epigenome. Nat. Neurosci. *20*, 1418.

Patro, R., Duggal, G., Love, M.I., Irizarry, R.A., and Kingsford, C. (2017). Salmon provides fast and bias-aware quantification of transcript expression. Nat. Methods *14*, 417–419.

Schroeder, A., Mueller, O., Stocker, S., Salowsky, R., Leiber, M., Gassmann, M., Lightfoot, S., Menzel, W., Granzow, M., and Ragg, T. (2006). The RIN: An RNA integrity number for assigning integrity values to RNA measurements. BMC Mol. Biol. *7*, 3.

Wilson, R.S., Boyle, P.A., Yu, L., Barnes, L.L., Sytsma, J., Buchman, A.S., Bennett, D.A., and Schneider, J.A. (2015). Temporal course and pathologic basis of unawareness of memory loss in dementia. Neurology *85*, 984–991.
