# Supplementary material for: Tau Activates Transposable Elements in Alzheimer’s Disease
Source: Cell Rep. Author manuscript; Available in PMC 2018 Oct 11. (PMC6181645; doi:10.1016/j.celrep.2018.05.004)
Supplement: 4 [file NIHMS1506631-supplement-4.pdf]

Cell Reports

Report

Tau Activates Transposable Elements in Alzheimer's Disease

Graphical Abstract

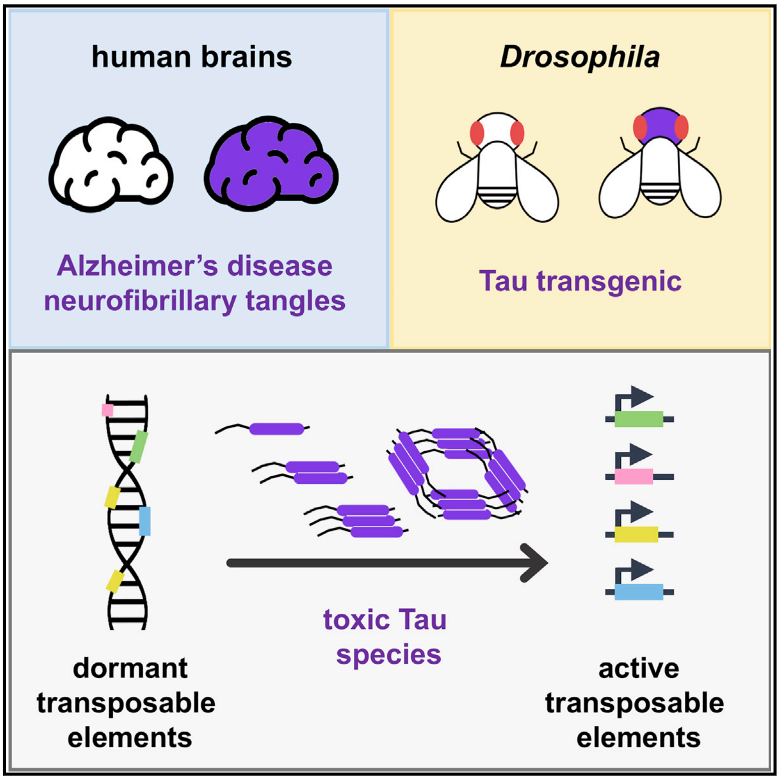

Highlights

- Alzheimer's Tau pathology is related to retrotransposon expression in human brains
- Neurofibrillary tangles also associate with chromatin relaxation at selected loci
- Tau activates transposable element transcription in transgenic flies

Authors

Caiwei Guo, Hyun-Hwan Jeong, Yi-Chen Hsieh, ..., Philip L. De Jager, Zhandong Liu, Joshua M. Shulman

Correspondence

joshua.shulman@bcm.edu

In Brief

Integrating studies of human postmortem brain tissue and *Drosophila melanogaster* models, Guo et al. show that Alzheimer's disease Tau neurofibrillary tangle pathology activates transcription of transposable element loci. An altered retrotransposon transcriptional landscape and associated genomic instability are implicated in Tau-mediated neurodegenerative mechanisms.

Guo et al., 2018, Cell Reports *23*, 2874–2880
June 5, 2018 © 2018 The Author(s).
https://doi.org/10.1016/j.celrep.2018.05.004

CellPress

OPEN ACCESS
CellPress

Cell Reports
Report

Tau Activates Transposable Elements in Alzheimer's Disease

Caiwei Guo,[1,2,9] Hyun-Hwan Jeong,[2,3,9] Yi-Chen Hsieh,[2,3] Hans-Ulrich Klein,[4,5] David A. Bennett,[6] Philip L. De Jager,[4,5] Zhandong Liu,[2,7] and Joshua M. Shulman[1,2,3,8,10,*]
[1]Department of Neuroscience, Baylor College of Medicine, Houston, TX 77030, USA
[2]Jan and Dan Duncan Neurologic Research Institute, Texas Children's Hospital, Houston, TX 77030, USA
[3]Department of Molecular and Human Genetics, Baylor College of Medicine, Houston, TX 77030, USA
[4]Center for Translational and Computational Neuroimmunology, Department of Neurology, Columbia University Medical Center, New York, NY 10032, USA
[5]Cell Circuits Program, Broad Institute, Cambridge, MA 02142, USA
[6]Rush Alzheimer's Disease Center, Rush University Medical Center, Chicago, IL 60612, USA
[7]Department of Pediatrics, Baylor College of Medicine, Houston, TX 77030, USA
[8]Department of Neurology, Baylor College of Medicine, Houston, TX 77030, USA
[9]These authors contributed equally
[10]Lead Contact
*Correspondence: joshua.shulman@bcm.edu
https://doi.org/10.1016/j.celrep.2018.05.004

SUMMARY

Aging and neurodegenerative disease are characterized by genomic instability in neurons, including aberrant activation and mobilization of transposable elements (TEs). Integrating studies of human postmortem brain tissue and *Drosophila melanogaster* models, we investigate TE activation in association with Tau pathology in Alzheimer's disease (AD). Leveraging RNA sequencing from 636 human brains, we discover differential expression for several retrotransposons in association with neurofibrillary tangle burden and highlight evidence for global TE transcriptional activation among the long interspersed nuclear element 1 and endogenous retrovirus clades. In addition, we detect Tau-associated, active chromatin signatures at multiple *HERV-Fc1* genomic loci. To determine whether Tau is sufficient to induce TE activation, we profile retrotransposons in *Drosophila* expressing human wild-type or mutant Tau throughout the brain. We discover heterogeneous response profiles, including both age- and genotype-dependent activation of TE expression by Tau. Our results implicate TE activation and associated genomic instability in Tau-mediated AD mechanisms.

INTRODUCTION

Alzheimer's disease (AD) is the most common neurodegenerative disorder and the leading cause of dementia, with more than 13 million individuals projected to be affected in the United States by 2050 (Querfurth and LaFerla, 2010). At autopsy, AD is characterized by extracellular neuritic plaques and intracellular neurofibrillary tangles, comprised of aggregated, misfolded amyloid-β peptide and Tau protein, respectively. Tau pathology is also found in a heterogeneous group of neurodegenerative syndromes, the tauopathies, causing cognitive and/or motor impairment. Based on evidence from human postmortem material (Adamec et al., 1999) and animal models (Khurana et al., 2012), AD brain pathology is accompanied by genomic instability in affected neurons (Madabhushi et al., 2014), and Tau-mediated mechanisms are strongly implicated. In *Drosophila*, Tau induces global nuclear chromatin relaxation (Frost et al., 2014), abnormal transcriptional activation of heterochromatic genes, and DNA double-strand breaks (Khurana et al., 2012). Importantly, genetic manipulation of chromatin-modifying or DNA-repair pathways can suppress Tau neurotoxicity, suggesting that the maintenance of genomic integrity and neurodegeneration in AD may be causally linked rather than simply a downstream consequence of cell death.

Transposable elements (TEs) are mobile genetic sequences present in all eukaryotic genomes examined to date (Levin and Moran, 2011). Although TE-derived sequences are estimated to account for ~45% of the human genome, the majority are degenerate and incapable of mobilization. However, somatic transposition of TEs—specifically retrotransposons, which mobilize through an RNA intermediate—has been documented in adult neurons, including in human brains (Baillie et al., 2011; Evrony et al., 2012; Upton et al., 2015) and both mouse (Muotri et al., 2005) and fly models (Perrat et al., 2013). TE activation may be harmful, potentially disrupting the transcriptional landscape and triggering an immunologic response (Kassiotis and Stoye, 2016). With TE mobilization, somatic insertional mutagenesis and genomic rearrangements may also occur. Numerous systems have therefore evolved to suppress TE activity, and these mechanisms overlap with those regulating chromatin structure and DNA repair (Levin and Moran, 2011). However, TE surveillance may deteriorate with brain aging, leading to retrotransposon activation (Li et al., 2013; Maxwell et al., 2011; Wood et al., 2016). Based on studies in humans and animal

This is an open access article under the CC BY license (http://creativecommons.org/licenses/by/4.0/).

Table 1. TEs Significantly Associated with Human Brain Tau Pathologic Burden

			Tangles		Plaques	
TE	Class	Clade	β	p Value	β	p Value
AluYh9	non-LTR	SINE	−0.033	3.34×10^{-6}	−0.023	1.10×10^{-5}
L1MB4_5	non-LTR	L1	0.032	1.43×10^{-4}	0.008	0.20
AluSp	non-LTR	SINE	−0.024	3.66×10^{-4}	−0.006	0.22
HERV-Fc1	LTR	ERV1	0.027	4.72×10^{-4}	0.011	0.05
AluYc5	non-LTR	SINE	0.033	5.17×10^{-4}	0.007	0.33
THER2	non-LTR	SINE	0.053	5.30×10^{-4}	0.021	0.08
PRIMA4_LTR	LTR	ERV1	0.025	6.65×10^{-4}	0.016	4.82×10^{-3}
LTR77	LTR	ERV1	0.026	8.14×10^{-4}	0.007	0.26
PB1D11	non-LTR	SINE	0.022	9.51×10^{-4}	0.013	7.39×10^{-3}

Top-ranked associations with tangle pathology are shown (false discovery rate < 0.1); comprehensive results for 547 retrotransposons are shown in Table S5. Only a subset of TEs was associated with amyloid plaques. β, beta coefficient.

models, aberrant TE activation has been implicated in many neurologic disorders, including multiple sclerosis (Morandi et al., 2017), Rett syndrome (Muotri et al., 2010), amyotrophic lateral sclerosis (ALS)-frontotemporal degeneration (FTD) (Douville et al., 2011; Li et al., 2015; Prudencio et al., 2017), and ataxia telangiectasia (Coufal et al., 2011). Evidence strongly suggests TEs may directly promote neuronal dysfunction and/or loss (Krug et al., 2017; Tan et al., 2012). For example, the RNA-binding protein, TDP-43, which aggregates in FTD-ALS, regulates the expression of TE transcripts (Li et al., 2012; Saldi et al., 2014), and inhibition of TE activation attenuates TDP-43 toxicity in fly models (Krug et al., 2017). Moreover, expression of the endogenous retrovirus (*HERV-K*) has been demonstrated in human cortical and spinal neurons in ALS, and the encoded Envelope (Env) protein is neurotoxic (Li et al., 2015).

Despite the evidence for genomic instability, retrotransposon expression has not been systematically evaluated in AD. In one small study, no differences in L1 genomic copy number were detected based on targeted PCR (Protasova et al., 2017). Here, we couple analyses of more than 600 human cortical transcriptomes with experiments in *Drosophila* transgenic models, highlighting global TE activation in AD and implicating Tau-mediated mechanisms.

RESULTS

Tau Pathologic Burden Is Associated with Altered TE Expression in Human Brains

To examine whether AD neurofibrillary tangle pathology is associated with TE activation, we first leveraged data from 2 prospective human clinical-pathologic studies, the Religious Orders Study and Rush Memory and Aging Project (ROSMAP). Our analyses included 636 deceased subjects with completed brain autopsies along with transcriptomic profiling of the dorsolateral prefrontal cortex based on RNA sequencing (RNA-seq). Clinical and demographic characteristics of our study cohort are detailed in Table S1. Current algorithms for building transcriptomes rely on alignment of RNA-seq data to a genomic reference, in which most repetitive sequences derived from TEs are excluded. In order to derive genome-wide estimates of transcription at TE loci in a computational efficient manner, we developed a tool, SalmonTE (Jeong et al., 2018). Based on a consensus TE sequence library from Repbase (Bao et al., 2015), we estimated transcriptional signatures for 366 long terminal repeat (LTR) and 181 non-LTR retrotransposons (Figure S1). We next applied linear regression, relating TE count estimates to a quantitative measure of average tangle burden, based on histologic counts from brain tissue sections. Table 1 highlights the 9 TEs significantly associated with Tau pathologic burden. Most TEs showed increased transcriptional activation, including selected long interspersed nuclear element 1 (LINE1 or L1), short interspersed nuclear elements (SINEs), and endogenous retroviruses (ERVs). To address specificity, we next examined each of the top-ranked TEs for associations with neuritic amyloid plaque pathology (Tables 1 and S3). Only a subset of the TE expression signatures was also associated with neuritic plaques, and the significance was attenuated. Given the small effect sizes and the large number of TEs, statistical power may be limited to detect associations for discrete TE expression signatures. We therefore performed a complementary analysis in which retrotransposons were aggregated based on clade membership, and the within-group distributions of t-statistic values for the association of TEs with tangles were evaluated. Interestingly, the ERV1, 2, 3, and L1 retrotransposon clades showed significant, positive deviation from the null distribution, consistent with global activation in the context of neurofibrillary tangle pathology (Figure 1; Table S2). Consistent TE clade activation patterns were also associated with AD pathologic diagnosis, and activation of the three ERV clades was related to global cognitive performance in the year proximate to death (Table S2). Our results suggest that the activity of TE loci may be broadly impacted by AD Tau pathology in human brains.

As introduced above, Tau pathology is associated with global chromatin reorganization and dysregulated gene expression (Frost et al., 2014; Klein et al., 2018). We hypothesized that Tau-induced chromatin relaxation might also de-repress silenced TEs. Among those elements implicated (Table 1), we focused on *HERV-Fc1*, which is unique for being present at low genomic copy number (n = 19 sites based on Dfam; Hubley et al., 2016). We leveraged an available chromatin immunoprecipitation sequencing (ChIP-seq) dataset, including 675 ROSMAP cortical samples, and extracted reads mapped to

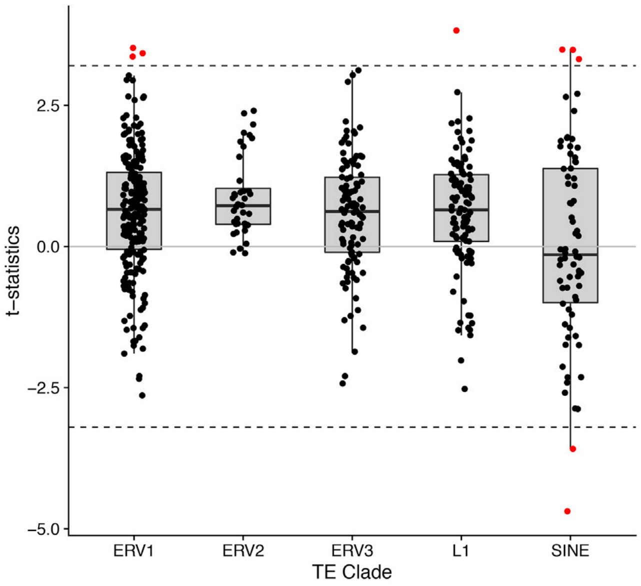

Figure 1. Tau Pathologic Burden Is Associated with Increased TE Expression in Human Brains

Boxplots display regression t-statistics aggregated based on TE clade annotations. The dotted lines indicate the significance threshold, denoting those TEs (red) with the most extreme associations listed in Table 1. The mean t-statistic was significantly inflated for L1 ($p = 7.1 \times 10^{-8}$), ERV1 ($p = 6.9 \times 10^{-14}$), ERV2 ($p = 1.9 \times 10^{-9}$), and ERV3 ($p = 8.2 \times 10^{-8}$), consistent with a global impact of Tau pathologic burden on TE expression. See also Table S2.

each *HERV-Fc1* genomic locus. Regression was performed to evaluate associations between tangle burden and level of histone 3, lysine 9 acetylation (H3K9Ac). Indeed, positive associations (p values < 0.05), indicating enhanced H3K9Ac, were detected at 4 out of 13 regions with available data; 3 regions remained significant after adjustment for multiple testing (Table S4). Our results support a hypothetical causal chain, in which Tau pathology promotes chromatin relaxation and TE transcriptional activation.

Tau Is Sufficient to Alter TE Activity in the *Drosophila* Adult Nervous System

In order to determine whether Tau pathology can induce activity of TE genomic loci, we turned to an established fly model relevant to AD (Wittmann et al., 2001). Pan-neuronal expression of either the wild-type human *MAPT* gene (Tau^{WT}) or a mutant form associated with familial FTD (Tau^{R406W}) causes age-dependent neuronal loss in association with hyperphosphorylated, misfolded Tau protein in the adult fly brain. We initially profiled 12 *Drosophila* TEs, including 8 LTR retrotransposons and 4 non-LTR retrotransposons, which have been previously demonstrated to be active in the fly nervous system, either following aging and/or manipulation of TE surveillance mechanisms (Li et al., 2013; Perrat et al., 2013). Tau^{WT}, Tau^{R406W}, or control flies were aged, and qRT-PCR was performed to assess TE transcriptional activity in adult heads. Three out of 12 TEs were significantly increased in one or both of the Tau transgenic lines at 20 days (Figure 2A), including both LTR (*copia* and *gypsy*) and L1-like, non-LTR retrotransposons (*het-a*). Notably, the *gypsy* TE is an endogenous insect retrovirus (Marlor et al., 1986) similar to human ERVs, including *HERV-Fc1*. The *Drosophila* retroelements were each increased ~3- to 10-fold in Tau transgenic flies compared with age-matched controls. Tau-dependent TE activation was already apparent in 1-day-old animals (Figure S2A), and for *copia*, expression increased progressively with aging (Figure 2B). Whereas *copia* and *het-a* showed evidence of enhanced activation in Tau^{R406W} animals, consistent with the increased toxicity of mutant Tau, expression of the *gypsy* TE was selectively increased in Tau^{WT} animals. Several other TEs were either unaffected or showed modest Tau-dependent reductions in expression when compared to age-matched control animals (Figures 2A and S2). Our results indicate that Tau is sufficient for activating expression of several *Drosophila* TEs in neurons but that the response profile is dependent on Tau genotype (wild-type versus mutant), aging, and the specific element examined. These findings were confirmed using an available RNA-seq dataset, permitting comprehensive assessments of TE expression signatures in 20-day-old Tau^{WT} versus control flies (Table S6). Our results reveal significant Tau-triggered expression changes affecting 64 out of 162 total retroelement signatures assayed (40%), including *gypsy*, *copia*, and *het-a* along with many other TEs. Out of the 37 Gypsy-class TE differential expression signatures, 22 (59%) demonstrated positive changes consistent with Tau-dependent activation (mean: 2.0; range: 1.3- to 26.6-fold increase). As a further control, the Tau^{WT} and Tau^{R406W} strains were each independently backcrossed to w^{1118} controls for 5 generations, ensuring a homogeneous genetic background. qPCR confirmed Tau-dependent increases in *het-a*, *copia*, and *gypsy* expression in aged animals (Figure S2C). Interestingly, we also detected modest but significant Tau- and age-dependent TE copy number increases based on qPCR of genomic DNA prepared from adult fly heads (Figure S2D), potentially consistent with retrotransposition (see below).

DISCUSSION

Our results, based on a cross-species strategy, implicate an altered TE transcriptional landscape in the setting of AD. Analyses of human brain transcriptomes identify differential retrotransposon expression signatures in association with neurofibrillary tangle burden along with evidence for widespread activation of selected TE clades, including L1 and the ERVs. L1 retrotransposons have previously been found to be activated in Rett syndrome (Muotri et al., 2010) and ataxia telangiectasia (Coufal et al., 2011), and ERV induction has been associated with ALS (Douville et al., 2011; Li et al., 2015) and multiple sclerosis (Morandi et al., 2017), including the same *HERV-Fc1* element detected in our analysis. We further discovered evidence of Tau-associated, active chromatin marks at genomic sites known to harbor *HERV-Fc1*. However, studies of human postmortem data in isolation are unable to establish causation. Moreover, Tau pathology co-exists in AD with neuritic amyloid plaques and other age-related brain lesions (Kapasi et al., 2017), making it difficult to establish specificity. We therefore turned to *Drosophila* transgenic models, revealing that Tau is sufficient to activate numerous TEs. For selected retrotransposons,

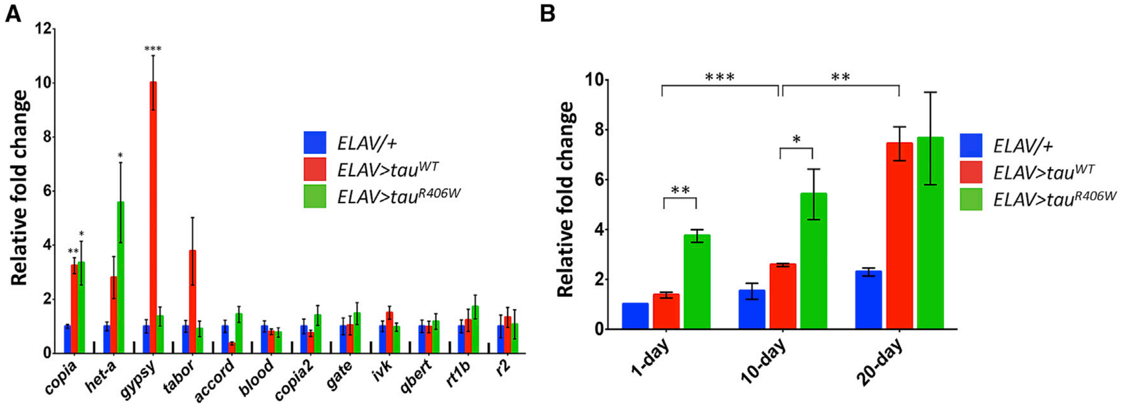

Figure 2. Tau Activates Expression of Selected TEs in the *Drosophila* Brain

(A) In 20-day-old animals, the *copia*, *het-a*, and *gypsy* retrotransposons were activated following neuronal expression of wild-type and/or mutant human Tau. Expression of 12 TEs was profiled by qPCR in fly heads from the following genotypes: (1) *ELAV-GAL4/+*; (2) *ELAV-GAL4/+; UAS-Tau^{WT}/+*; and (3) *ELAV-GAL4/+; UAS-Tau^{R406W}/+*. One-way ANOVA model F-test was significant ($p < 0.05$) for *copia*, *het-a*, and *gypsy*. Analyses of 1- and 10-day-old animals are shown in Figure S2.

(B) Expression of the *copia* retrotransposon is enhanced by age and mutant Tau. Two-way ANOVA testing was significant ($p < 0.0001$) for both age and genotype. All results (A and B) were normalized to *RpL32* expression, and fold-change relative to 1-day-old *ELAV-GAL4/+* control flies is shown (mean ± SEM). Subsetted t tests were performed for post hoc comparisons. *$p < 0.05$; **$p < 0.01$; ***$p < 0.001$.

activation was further enhanced with aging and by a mutant form of Tau associated with increased neurotoxicity. We propose a model (Figure 3) in which Tau modulates transcriptional activity at TE loci, possibly via chromatin remodeling, leading to neuronal dysfunction and/or loss. It is likely that other brain pathologies besides tangles also contribute to TE activation in AD. In our analyses, TE expression was also associated—albeit more weakly—with neuritic amyloid plaque pathologic burden. TDP-43 pathology, which commonly occurs in brains affected by AD (Kapasi et al., 2017), has also been associated with TE activation in human brains (Li et al., 2012, 2015) and fly models (Krug et al., 2017).

TE activation might both arise from and further promote genomic instability in AD. In both human brains and animal models, AD pathologic changes have been associated with epigenomic remodeling and transcriptional dysregulation (Frost et al., 2014; Gjoneska et al., 2015; De Jager et al., 2014). In ROSMAP, Tau pathology was related to widespread alterations in histone acetylation and similar changes were not associated with amyloid-β pathology (Klein et al., 2018). Expression of human Tau in the *Drosophila* brain also causes global chromatin relaxation and aberrant transcriptional activation of many genes that are usually repressed in heterochromatin (Frost et al., 2014). This suggests a potential mechanism for TE activation, because maintenance of these loci in a transcriptionally silent, heterochromatic state is one important mechanism for TE suppression (Levin and Moran, 2011). Based on public databases, the TEs most strongly implicated in our analyses (Table 1) are highly duplicated in the human genome. Moreover, our RNA-seq-based alignment strategy does not permit definitive localization of the genomic site(s) of origin for TE transcriptional activation. For example, the top-ranked *AluYh9* SINE element maps to 392 distinct genomic positions. For *HERV-Fc1*, which is present at comparatively low copy number, we provide additional evidence in support of our model, highlighting H3K9Ac chromatin marks at several loci consistent with active and/or relaxed chromatin conformation and resulting transcriptional activation. Importantly, genetic manipulations that restore chromatin packing in *Drosophila* have been demonstrated to rescue neuronal loss (Frost et al., 2014), indicating that these changes are causally linked to Tau-mediated neurodegeneration.

Aberrant TE expression may be highly damaging to neurons (Figure 3). Beyond the potential for TE mobilization (see below), with associated genomic rearrangements and insertional mutagenesis, even isolated transcriptional activation could be harmful. Both the innate and adaptive arms of the immune system can recognize retrotransposon-derived transcripts and/or proteins, provoking potent neuroinflammatory responses (Kassiotis and Stoye, 2016). Because the majority of TEs in the human genome have internal deletions or mutations that render them incompetent for autonomous mobilization, aberrant expression and activation of endogenous immunologic surveillance may be the most important implication of our findings. Indeed, TE-triggered immune reactions have been suggested in both multiple sclerosis (Antony et al., 2004) and ALS (Douville et al., 2011). In age-related macular degeneration, the accumulation of SINE *Alu* RNAs has been linked to an innate immune response that causes degeneration of the retinal pigment epithelium and resulting blindness (Tarallo et al., 2012). Neuroinflammation has also been implicated in AD pathogenesis, where the inciting events remain incompletely defined (Heppner et al., 2015).

One limitation of our study design is that we are unable to directly address whether retrotransposons mobilize causing new genomic insertions. In *Drosophila*, TEs can mobilize in the adult brain (Perrat et al., 2013), and *de novo* L1 insertions have been similarly documented in human brains and neural progenitor cells (Baillie et al., 2011; Coufal et al., 2009; Evrony et al., 2012; Upton et al., 2015). Our analyses suggest global activation of L1 elements in AD (Figure 1). However, of the 9 discrete TEs showing significant Tau-associated expression changes

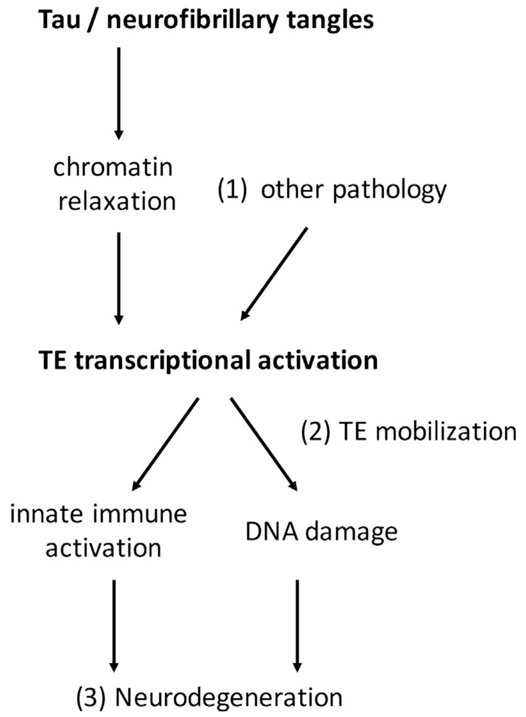

Figure 3. Hypothetical Model and Remaining Questions
Our results along with other published evidence inform a causal model for Tau-mediated TE activation in AD, along with key knowledge gaps for further investigation (1–3). Tau is sufficient to induce TE transcriptional activation. Analyses of *HERV-Fc1* suggest that chromatin changes may in part be responsible, but other mechanisms may also contribute. (1) Besides Tau, it is likely that additional brain pathologies promote TE activation. (2) It remains to be determined whether TE transcriptional activation in AD leads to mobilization, potentially contributing to DNA damage and genomic instability. In the absence of transposition, TE expression may provoke an innate immune response. (3) Although DNA damage and neuroinflammation are strongly implicated in AD neurodegeneration, additional studies will be required to assess whether TEs contribute.

(Table 1), none are predicted to be capable of autonomous transposition. For example, based on Repbase, *L1MB4_5* is a "degenerate" L1 element lacking an intact open reading frame 2, which encodes a protein (ORF2p) with reverse transcriptase and endonuclease activity. Notably, most L1 retrotransposition in the human genome is believed to be due to the evolutionary young L1H subfamily, and such elements were absent from our top hits (Brouha et al., 2003). We also identify Tau-associated changes in the expression of several SINE retrotransposons. Although not capable of autonomous replication, SINE transposition can be facilitated by ORF2p provided by an active L1 element. Of the implicated SINEs, *AluYc5* is an evolutionary young Alu element, which is among the most abundant and active TEs in the human genome (Deininger, 2011). *HERV-Fc1* is an LTR retrotransposon including a *gag*, *pol*, and *env* gene flanked by two LTRs, but only the *env* open reading frame is known to be intact (Bénit et al., 2003). Selective expression of the Env protein from the related *HERV-K* is neurotoxic in human neuronal cultures and mouse models (Li et al., 2015), consistent with a similar model in AD, in which TE expression, but not mobilization, may be sufficient for neuronal injury. Our finding of Tau-induced increases in genomic copy number for the *copia*, *gypsy*, and *het-a* TEs in flies (Figure S2) is potentially compatible with retrotransposition; however, this evidence is indirect and should be interpreted cautiously because alternative explanations are possible (e.g., amplification of existing genomic loci). Definitive evidence of Tau-induced TE transposition will require genomic sequencing to identify potential new insertion sites.

Prior investigations in both animal models and human postmortem tissue have documented DNA double-strand breaks and activation of repair pathways in association with AD pathologic changes (Madabhushi et al., 2014). Whereas numerous triggers have been proposed, including oxidative injury, neuronal excitability, and chromatin disruption, TE activation and/or mobilization may also contribute to genomic instability in AD (Figure 3). There is significant overlap between regulators of the DNA damage response and TE surveillance mechanisms (Levin and Moran, 2011). Importantly, manipulation of many such factors, including *loki* (homolog of human *CHEK2*), *atm*, *p53*, and *Ago3*, are potent modulators of Tau-induced neurodegenerative phenotypes in *Drosophila* (Khurana et al., 2012; Frost et al., 2014). In future work, it will be essential to determine whether TEs are causally linked to DNA damage and genomic instability in AD and whether this contributes to neurodegeneration.

EXPERIMENTAL PROCEDURES

For detailed methods, see Supplemental Experimental Procedures.

Analysis of TE Expression from Human Brain Transcriptomes

ROSMAP participants were free of known dementia at enrollment, agreed to annual clinical evaluations, and signed an informed consent and Anatomic Gift Act donating their brains at death, approved by the Institutional Review Board at Rush University. The modified Bielschowsky silver stain was used to visualize and quantify neurofibrillary tangles and neuritic plaques. ROSMAP RNA-seq data were generated with Illumina Hi-Seq. TE expression signatures were estimated using SalmonTE (Jeong et al., 2018) and a reference library derived from Repbase (Bao et al., 2015; Figure S1A). In selected cases, TE expression was independently confirmed by PCR (Figure S1B). Linear regression was performed to examine the relation of each TE expression signature (batch-corrected, log-transformed transcripts per million) with the neurofibrillary tangle burden outcome, adjusting for age at death, postmortem interval (PMI), and RNA integrity number. Statistical significance was based on a Benjamini-Hochberg false discovery rate (FDR) < 0.1. In order to evaluate associations for each TE clade, t-statistic values were aggregated from our primary regression model (Table S5), and a one-sample t test was performed to evaluate for a non-zero mean t-statistic value (Figure 1; Table S2). For the analyses of chromatin remodeling at *HERV-Fc1* loci, a log-linear regression model was implemented to evaluate associations between neurofibrillary tangle burden and H3K9Ac ChIP-seq reads, adjusting for library size, batch, PMI, cross-correlation, age, and gender.

Analysis of TE Expression in *Drosophila* Heads

The *UAS-Tau*^{WT} and *UAS-Tau*^{R406W} transgenic flies were previously described (Wittmann et al., 2001). The following genotypes were used: (1) *ELAV-GAL4/+* (control); (2) *ELAV-GAL4/+; UAS-Tau*^{WT}*/+*; and (3) *ELAV-GAL4/+; UAS-Tau*^{R406W}*/+*. qPCR was performed from total RNA prepared from fly heads in triplicate samples, using *RpL32* as an internal control. Expression ($\Delta\Delta C_T$ values) was normalized to that of 1-day-old control animals. One-way ANOVA was performed to detect differences between group mean expression values, considering each TE and time point separately. Subsetted t tests (two-tailed) were subsequently performed for post hoc comparisons of each Tau genotype with control animals. For *copia*, two-way ANOVA was secondarily

performed to differentiate genotype and age effects. Error bars in all analyses represent the SEM. RNA-seq data from *ELAV-GAL4/+; UAS-Tau^{WT}/+* or control (*ELAV-GAL4/+*) fly heads were analyzed using SalmonTE. Statistical significance was set to FDR < 0.1. For analyses of TE copy number, genomic DNA was extracted from heads of 10-day-old flies prior to qPCR.

DATA AND SOFTWARE AVAILABILITY

The following datasets are available at the indicated websites: ROSMAP RNA-seq: https://doi.org/10.7303/syn3388564; ROSMAP ChIP-seq: https://doi.org/10.7303/syn4896408; and *Drosophila* RNA-seq: https://doi.org/10.7303/syn7274101. Additional data from the ROSMAP study are available on the RADC Research Resource Sharing Hub at http://www.radc.rush.edu/. The SalmonTE software tool is available for download at https://github.com/hyunhwaj/SalmonTE.

SUPPLEMENTAL INFORMATION

Supplemental Information includes Supplemental Experimental Procedures, two figures, and six tables and can be found with this article online at https://doi.org/10.1016/j.celrep.2018.05.004.

ACKNOWLEDGMENTS

This study was supported by grants from the NIH (R01AG053960, R01AG050631, U01AG046161, R01AG033193, C06RR029965, P30AG10161, R01AG15819, R01AG17917, U01AG46152, R01AG36836, R01GM120033, R01AG36042, and RC236547), the Alzheimer's Association, the American Federation for Aging Research, Huffington Foundation, Jan and Dan Duncan Neurological Research Institute at Texas Children's Hospital, the Burroughs Wellcome Fund, the National Science Foundation (DMS-1263932), Cancer Prevention Research Institute of Texas (RP170387), Houston Endowment, and the Belfer Neurodegenerative Disease Consortium. We thank Nikolaos Giagtzoglou, Martin Powers, and Marissa Scavuzzo for helpful discussions.

AUTHOR CONTRIBUTIONS

Conceptualization, J.M.S. and Z.L.; Methodology, H.-H.J. and Z.L.; Investigation, C.G. and Y.-C.H.; Formal Analysis, H.-H.J. and H.-U.K.; Resources, P.L.D.J. and D.A.B.; Writing – Original Draft, C.G., H.-H.J., H.-U.K., and J.M.S.; Writing – Review & Editing, Z.L., P.L.D.J., D.A.B., and J.M.S.; Supervision, J.M.S. and Z.L.; Funding Acquisition, J.M.S., Z.L., P.L.D.J., and D.A.B.

DECLARATION OF INTERESTS

The authors declare no competing interests.

Received: September 22, 2017
Revised: March 19, 2018
Accepted: May 1, 2018
Published: June 5, 2018

REFERENCES

Adamec, E., Vonsattel, J.P., and Nixon, R.A. (1999). DNA strand breaks in Alzheimer's disease. Brain Res. *849*, 67–77.

Antony, J.M., van Marle, G., Opii, W., Butterfield, D.A., Mallet, F., Yong, V.W., Wallace, J.L., Deacon, R.M., Warren, K., and Power, C. (2004). Human endogenous retrovirus glycoprotein-mediated induction of redox reactants causes oligodendrocyte death and demyelination. Nat. Neurosci. *7*, 1088–1095.

Baillie, J.K., Barnett, M.W., Upton, K.R., Gerhardt, D.J., Richmond, T.A., De Sapio, F., Brennan, P.M., Rizzu, P., Smith, S., Fell, M., et al. (2011). Somatic retrotransposition alters the genetic landscape of the human brain. Nature *479*, 534–537.

Bao, W., Kojima, K.K., and Kohany, O. (2015). Repbase Update, a database of repetitive elements in eukaryotic genomes. Mob. DNA *6*, 11.

Bénit, L., Calteau, A., and Heidmann, T. (2003). Characterization of the low-copy HERV-Fc family: evidence for recent integrations in primates of elements with coding envelope genes. Virology *312*, 159–168.

Brouha, B., Schustak, J., Badge, R.M., Lutz-Prigge, S., Farley, A.H., Moran, J.V., and Kazazian, H.H., Jr. (2003). Hot L1s account for the bulk of retrotransposition in the human population. Proc. Natl. Acad. Sci. USA *100*, 5280–5285.

Coufal, N.G., Garcia-Perez, J.L., Peng, G.E., Yeo, G.W., Mu, Y., Lovci, M.T., Morell, M., O'Shea, K.S., Moran, J.V., and Gage, F.H. (2009). L1 retrotransposition in human neural progenitor cells. Nature *460*, 1127–1131.

Coufal, N.G., Garcia-Perez, J.L., Peng, G.E., Marchetto, M.C.N., Muotri, A.R., Mu, Y., Carson, C.T., Macia, A., Moran, J.V., and Gage, F.H. (2011). Ataxia telangiectasia mutated (ATM) modulates long interspersed element-1 (L1) retrotransposition in human neural stem cells. Proc. Natl. Acad. Sci. USA *108*, 20382–20387.

De Jager, P.L., Srivastava, G., Lunnon, K., Burgess, J., Schalkwyk, L.C., Yu, L., Eaton, M.L., Keenan, B.T., Ernst, J., McCabe, C., et al. (2014). Alzheimer's disease: early alterations in brain DNA methylation at ANK1, BIN1, RHBDF2 and other loci. Nat. Neurosci. *17*, 1156–1163.

Deininger, P. (2011). Alu elements: know the SINEs. Genome Biol. *12*, 236.

Douville, R., Liu, J., Rothstein, J., and Nath, A. (2011). Identification of active loci of a human endogenous retrovirus in neurons of patients with amyotrophic lateral sclerosis. Ann. Neurol. *69*, 141–151.

Evrony, G.D., Cai, X., Lee, E., Hills, L.B., Elhosary, P.C., Lehmann, H.S., Parker, J.J., Atabay, K.D., Gilmore, E.C., Poduri, A., et al. (2012). Single-neuron sequencing analysis of L1 retrotransposition and somatic mutation in the human brain. Cell *151*, 483–496.

Frost, B., Hemberg, M., Lewis, J., and Feany, M.B. (2014). Tau promotes neurodegeneration through global chromatin relaxation. Nat. Neurosci. *17*, 357–366.

Gjoneska, E., Pfenning, A.R., Mathys, H., Quon, G., Kundaje, A., Tsai, L.H., and Kellis, M. (2015). Conserved epigenomic signals in mice and humans reveal immune basis of Alzheimer's disease. Nature *518*, 365–369.

Heppner, F.L., Ransohoff, R.M., and Becher, B. (2015). Immune attack: the role of inflammation in Alzheimer disease. Nat. Rev. Neurosci. *16*, 358–372.

Hubley, R., Finn, R.D., Clements, J., Eddy, S.R., Jones, T.A., Bao, W., Smit, A.F.A., and Wheeler, T.J. (2016). The Dfam database of repetitive DNA families. Nucleic Acids Res. *44* (*D1*), D81–D89.

Jeong, H.H., Yalamanchili, H.K., Guo, C., Shulman, J.M., and Liu, Z. (2018). An ultra-fast and scalable quantification pipeline for transposable elements from next generation sequencing data. Pac. Symp. Biocomput. *23*, 168–179.

Kapasi, A., DeCarli, C., and Schneider, J.A. (2017). Impact of multiple pathologies on the threshold for clinically overt dementia. Acta Neuropathol. *134*, 171–186.

Kassiotis, G., and Stoye, J.P. (2016). Immune responses to endogenous retroelements: taking the bad with the good. Nat. Rev. Immunol. *16*, 207–219.

Khurana, V., Merlo, P., DuBoff, B., Fulga, T.A., Sharp, K.A., Campbell, S.D., Götz, J., and Feany, M.B. (2012). A neuroprotective role for the DNA damage checkpoint in tauopathy. Aging Cell *11*, 360–362.

Klein, H.-U., McCabe, C., Gjoneska, E., Sullivan, S.E., Kaskow, B.J., Tang, A., Smith, R.V., Xu, J., Pfenning, A.R., Bernstein, B.E., et al. (2018). Epigenome-wide study uncovers tau pathology-driven changes of chromatin organization in the aging human brain. bioRxiv. https://doi.org/10.1101/273789.

Krug, L., Chatterjee, N., Borges-Monroy, R., Hearn, S., Liao, W.W., Morrill, K., Prazak, L., Rozhkov, N., Theodorou, D., Hammell, M., and Dubnau, J. (2017). Retrotransposon activation contributes to neurodegeneration in a Drosophila TDP-43 model of ALS. PLoS Genet. *13*, e1006635.

Levin, H.L., and Moran, J.V. (2011). Dynamic interactions between transposable elements and their hosts. Nat. Rev. Genet. *12*, 615–627.

Li, W., Jin, Y., Prazak, L., Hammell, M., and Dubnau, J. (2012). Transposable elements in TDP-43-mediated neurodegenerative disorders. PLoS ONE *7*, e44099.

Li, W., Prazak, L., Chatterjee, N., Grüninger, S., Krug, L., Theodorou, D., and Dubnau, J. (2013). Activation of transposable elements during aging and neuronal decline in Drosophila. Nat. Neurosci. *16*, 529–531.

Li, W., Lee, M.H., Henderson, L., Tyagi, R., Bachani, M., Steiner, J., Campanac, E., Hoffman, D.A., von Geldern, G., Johnson, K., et al. (2015). Human endogenous retrovirus-K contributes to motor neuron disease. Sci. Transl. Med. *7*, 307ra153.

Madabhushi, R., Pan, L., and Tsai, L.H. (2014). DNA damage and its links to neurodegeneration. Neuron *83*, 266–282.

Marlor, R.L., Parkhurst, S.M., and Corces, V.G. (1986). The Drosophila melanogaster gypsy transposable element encodes putative gene products homologous to retroviral proteins. Mol. Cell. Biol. *6*, 1129–1134.

Maxwell, P.H., Burhans, W.C., and Curcio, M.J. (2011). Retrotransposition is associated with genome instability during chronological aging. Proc. Natl. Acad. Sci. USA *108*, 20376–20381.

Morandi, E., Tanasescu, R., Tarlinton, R.E., Constantinescu, C.S., Zhang, W., Tench, C., and Gran, B. (2017). The association between human endogenous retroviruses and multiple sclerosis: a systematic review and meta-analysis. PLoS ONE *12*, e0172415.

Muotri, A.R., Chu, V.T., Marchetto, M.C.N., Deng, W., Moran, J.V., and Gage, F.H. (2005). Somatic mosaicism in neuronal precursor cells mediated by L1 retrotransposition. Nature *435*, 903–910.

Muotri, A.R., Marchetto, M.C.N., Coufal, N.G., Oefner, R., Yeo, G., Nakashima, K., and Gage, F.H. (2010). L1 retrotransposition in neurons is modulated by MeCP2. Nature *468*, 443–446.

Perrat, P.N., DasGupta, S., Wang, J., Theurkauf, W., Weng, Z., Rosbash, M., and Waddell, S. (2013). Transposition-driven genomic heterogeneity in the Drosophila brain. Science *340*, 91–95.

Protasova, M.S., Gusev, F.E., Grigorenko, A.P., Kuznetsova, I.L., Rogaev, E.I., and Andreeva, T.V. (2017). Quantitative analysis of L1-retrotransposons in Alzheimer's disease and aging. Biochemistry (Mosc.) *82*, 962–971.

Prudencio, M., Gonzales, P.K., Cook, C.N., Gendron, T.F., Daughrity, L.M., Song, Y., Ebbert, M.T.W., van Blitterswijk, M., Zhang, Y.J., Jansen-West, K., et al. (2017). Repetitive element transcripts are elevated in the brain of C9orf72 ALS/FTLD patients. Hum. Mol. Genet. *26*, 3421–3431.

Querfurth, H.W., and LaFerla, F.M. (2010). Alzheimer's disease. N. Engl. J. Med. *362*, 329–344.

Saldi, T.K., Ash, P.E., Wilson, G., Gonzales, P., Garrido-Lecca, A., Roberts, C.M., Dostal, V., Gendron, T.F., Stein, L.D., Blumenthal, T., et al. (2014). TDP-1, the Caenorhabditis elegans ortholog of TDP-43, limits the accumulation of double-stranded RNA. EMBO J. *33*, 2947–2966.

Tan, H., Qurashi, A., Poidevin, M., Nelson, D.L., Li, H., and Jin, P. (2012). Retrotransposon activation contributes to fragile X premutation rCGG-mediated neurodegeneration. Hum. Mol. Genet. *21*, 57–65.

Tarallo, V., Hirano, Y., Gelfand, B.D., Dridi, S., Kerur, N., Kim, Y., Cho, W.G., Kaneko, H., Fowler, B.J., Bogdanovich, S., et al. (2012). DICER1 loss and Alu RNA induce age-related macular degeneration via the NLRP3 inflammasome and MyD88. Cell *149*, 847–859.

Upton, K.R., Gerhardt, D.J., Jesuadian, J.S., Richardson, S.R., Sánchez-Luque, F.J., Bodea, G.O., Ewing, A.D., Salvador-Palomeque, C., van der Knaap, M.S., Brennan, P.M., et al. (2015). Ubiquitous L1 mosaicism in hippocampal neurons. Cell *161*, 228–239.

Wittmann, C.W., Wszolek, M.F., Shulman, J.M., Salvaterra, P.M., Lewis, J., Hutton, M., and Feany, M.B. (2001). Tauopathy in Drosophila: neurodegeneration without neurofibrillary tangles. Science *293*, 711–714.

Wood, J.G., Jones, B.C., Jiang, N., Chang, C., Hosier, S., Wickremesinghe, P., Garcia, M., Hartnett, D.A., Burhenn, L., Neretti, N., and Helfand, S.L. (2016). Chromatin-modifying genetic interventions suppress age-associated transposable element activation and extend life span in *Drosophila*. Proc. Natl. Acad. Sci. USA *113*, 11277–11282.

Cell Reports, Volume *23*

Supplemental Information

Tau Activates Transposable Elements in Alzheimer's Disease

Caiwei Guo, Hyun-Hwan Jeong, Yi-Chen Hsieh, Hans-Ulrich Klein, David A. Bennett, Philip L. De Jager, Zhandong Liu, and Joshua M. Shulman

SUPPLEMENTAL FIGURES

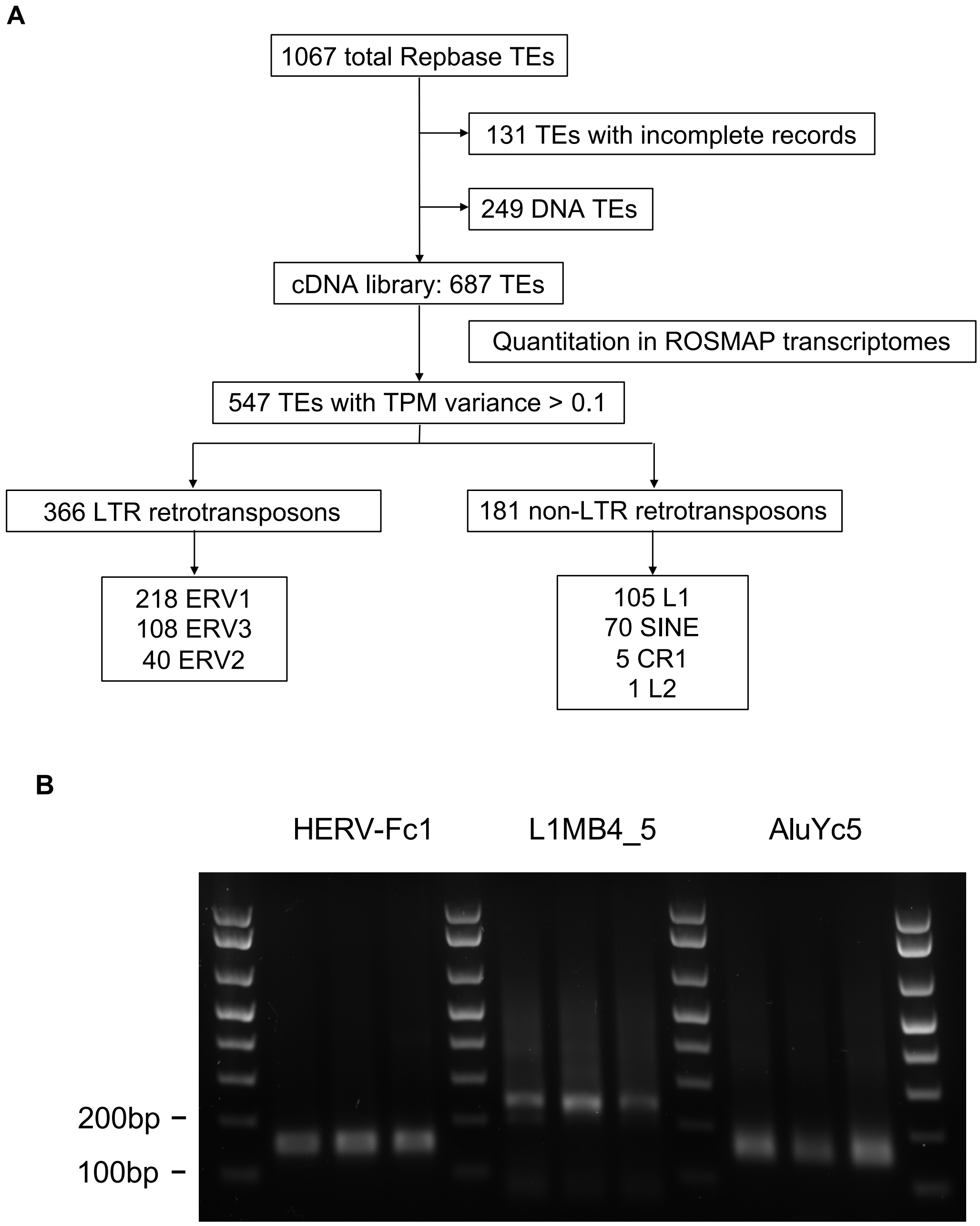

Figure S1. Profiling of TEs in human brain transcriptomes (Related to Figure 1).
(A) Schematic overview of the establishment of a human TE library for use in this study. (B) PCR validation of selected human TEs in 3 independent cortical RNA samples from ROSMAP postmortem brain tissue specimens. Agarose gel electrophoresis of PCR products for HERV-Fc1 (155bp), L1MB4_5 (219bp), and AluYc5 (142bp) are shown.

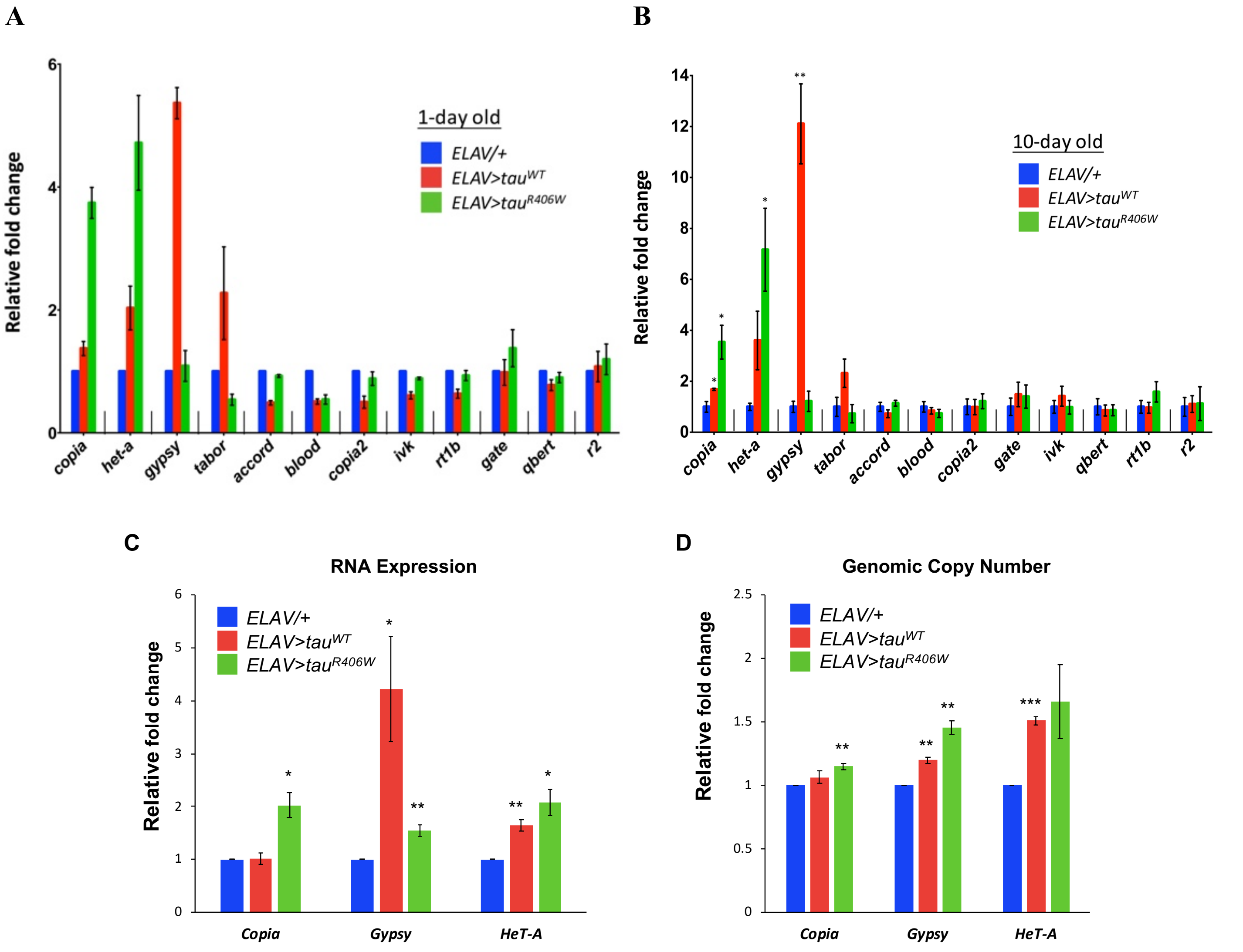

Figure S2. Tau activates TEs in the *Drosophila* brain (Related to Figure 2).
(A, B) Expression of 12 TEs was profiled by qPCR in triplicate samples from heads of *ELAV-GAL4/+*, *ELAV>Tau*^{WT}, and *ELAV>Tau*^{R406W} in 1-day-old (A) and 10-day-old (B) animals. Analyses conducted in 20-day-old animals are shown in Figure 2. (C, D) As a further control, the *UAS-Tau*^{WT} and *UAS-Tau*^{R406W} strains were back-crossed to w^{1118} control flies for 5 generations, ensuring a homogenous genetic background among our control and experimental genotypes. mRNA expression (C) or genomic copy number (D) of *copia*, *gypsy*, and *het-a* was profiled by qPCR in the heads of *ELAV-GAL4/+*, *ELAV>Tau*^{WT}, and *ELAV>Tau*^{R406W} in 10-day-old animals which were back crossed to w^{1118} flies for 5 generations. Since the 1-day old *ELAV-GAL4/+* control data was used for normalization, statistical analysis was not performed for comparisons at 1-day (A). For all other experiments (B-D), one-way ANOVA was performed to detect differences between group means, considering each TE separately (model F-test $p<0.05$ for *copia*, *het-a*, *gypsy*). Subsetted t-tests were subsequently performed for post-hoc comparisons of each Tau genotype with control animals. All results were normalized to *RpL32* and fold-change relative to *ELAV/+* control flies is shown (mean±S.E.M.). *, $p<0.05$, **, $p<0.01$, ***, $p<0.001$.

Table S1. ROSMAP autopsy cohort clinical and demographic characteristics (Related to Table 1).

**n**	636
**Age at enrollment, mean yr (SD)**	81.3 (7.0)
**Age at death, mean yr (SD)**	88.7 (6.7)
**Male, n**	229 (36.0%)
**Education, mean yr (SD)**	16.4 (3.5)
**No Cognitive Impairment, n**	201 (31.7%)
**Mild Cognitive Impairment, n**	168 (26.5%)
**Clinical AD, n**	254 (40.0%)
**Postmortem interval, mean hrs (SD)**	7.3 (4.8)
**Pathologic AD, n**	385 (60.5%)
**Neurofibrillary Tangle Burden, mean (SD)**	0.6 (0.8)
**Braak Score, n**	
**0**	7 (1.1%)
**I**	51 (8.0%)
**II**	54 (8.5%)
**III**	175 (27.5%)
**IV**	210 (33.0%)
**V**	132 (20.8%)
**VI**	7 (1.1%)

Clinical and pathologic diagnoses of AD were based on NINCDS-ADRDA and NIA-Reagan criteria, respectively. Neurofibrillary tangle pathology based on averaged pathologic counts from silver-stained tissue sections.

Table S2. TE clade associations with tangles, AD pathology, and cognition (Related to Figure 1).

		tangles		AD diagnosis		cognition	
**TE clade**	**n**	**mean (SE)**	**p-value**	**mean (SE)**	**p-value**	**mean (SE)**	**p-value**
ERV1	218	0.61 (0.08)	6.94×10^{-14}	0.50 (0.06)	1.45×10^{-14}	0.46 (0.07)	6.56×10^{-10}
ERV2	40	0.87 (0.11)	1.91×10^{-9}	0.62 (0.10)	2.35×10^{-7}	0.44 (0.12)	7.00×10^{-4}
ERV3	108	0.57 (0.10)	8.16×10^{-8}	0.47 (0.09)	5.12×10^{-7}	0.40 (0.11)	3.21×10^{-4}
L1	105	0.59 (0.10)	7.08×10^{-8}	0.28 (0.09)	1.18×10^{-3}	0.09 (0.09)	0.34
SINE	70	0.04 (0.21)	0.86	-0.02 (0.15)	0.97	-0.10 (0.19)	0.95

To evaluate summary associations for each TE clade, t-statistics were extracted from the regression model, and aggregated based on Repbase classifications. A one-sample t-test (two-tailed) was performed to evaluate for a non-zero mean t-statistic for each clade. Secondary analyses additionally examined associations between each TE clade and either consensus AD pathologic diagnosis (Bennett et al., 2006; National Institute on Aging, 1997) or global cognitive performance (Wilson et al., 2015), using logistic or linear regression, respectively. All analyses were adjusted for age at death, PMI and RIN, and the analysis for cognitive performance was additionally adjusted for sex and years of education. The mean t-statistic, standard error (SE), and p-value is shown for each TE clade. The results for neurofibrillary tangles correspond to the data presented in Figure 1.

Table S3. Alternate regression models to assess contributions of plaques and tangles (Related to Table 1).

TE	R-Squared (p-value)		
	**Model 1**	**Model 2**	**Model 3**
AluYh9	0.079 ($1.4x10^{-10}$)	0.076 ($4.31x10^{-10}$)	0.086 (5.27 $x10^{-11}$)
L1MB4_5	0.042 ($1.8x10^{-5}$)	0.023 ($6.05x10^{-3}$)	0.044 (3.39 $x10^{-5}$)
AluSp	0.060 ($6.4x10^{-8}$)	0.043 ($1.29x10^{-5}$)	0.061 (1.55 $x10^{-7}$)
HERV-Fc1	0.030 ($6.3x10^{-4}$)	0.017 (0.03)	0.030 (1.56 $x10^{-3}$)
AluYc5	0.025 ($3.2x10^{-3}$)	0.008 (0.31)	0.027 (4.39 $x10^{-3}$)
THER2	0.023 ($5.8x10^{-3}$)	0.009 (0.23)	0.023 (0.01)
PRIMA4_LTR	0.051 ($1.1x10^{-6}$)	0.046 ($5.94x10^{-6}$)	0.053 (1.85 $x10^{-6}$)
LTR77	0.028 ($1.3x10^{-3}$)	0.013 (0.09)	0.029 (2.31 $x10^{-3}$)
PB1D11	0.028 ($1.5x10^{-3}$)	0.021 ($7.78x10^{-3}$)	0.029 (2.19 $x10^{-3}$)

Alternative regression models were considered relating TE expression with AD pathology. All models were similarly adjusted for age, postmortem interval (PMI), and RNA integrity number (RIN). First, we examined the primary model (Model 1: TE ~ NFT + age + PMI + RIN) relating neurofibrillary tangle (NFT) pathology with TE expression signatures, noting the regression model R-squared and overall model F-test p-value. We next examined a similar adjusted model (Model 2: TE ~ NP + age + PMI + RIN), but considering the association with neuritic amyloid plaques (NP). Lastly, we considered a joint model (Model 3: TE ~ NFT + NP + age + PMI + RIN) including terms for both neuritic plaques and neurofibrillary tangles. The results reveal that including the neuritic plaque term explains little, if any, additional variance in TE expression beyond that already explained by neurofibrillary tangles. Only in 1 case (AluYh9) does the model p-value improve in the joint Model 3 compared Model 1.

Table S4. Tau-associated heterochromatin relaxation at *HERV-Fc1* loci (Related to Table 1).

Loci	Chr	StartPos	EndPos	Mean	SD	β	p-value
TE1	1	169685137	169688136	28.151	9.402	0.053	0.028
TE2	1	224886406	224889405	40.804	11.718	0.033	0.085
TE3	2	83874409	83879018	35.821	10.951	0.015	0.473
TE4	6	158884733	158887732	38.036	10.604	-0.012	0.502
TE5	7	64835238	64839812	167.942	40.821	0.006	0.579
TE6	7	153409350	153413807	19.483	6.507	0.036	0.142
TE7	11	5927657	5930656	20.893	7.200	-0.040	0.127
TE9	14	70297503	70300502	18.994	6.537	0.081	**0.001**
TE10	14	92046411	92049410	32.129	9.341	0.057	**0.004**
TE12	19	52754206	52757205	27.840	8.681	0.076	**0.001**
TE13	22	39059285	39062284	32.302	9.356	0.004	0.861
TE14	X	75967359	75970358	18.917	8.299	-0.006	0.832
TE15	Y	10050093	10053092	13.031	7.004	-0.007	0.823

HERV-Fc1 genomic loci were determined based on the Dfam database (Hubley et al., 2015). Coordinates are from GRCh37 (hg19) reference. A negative binomial regression model was fitted for each HERV-Fc1 locus to test the association between H3K9Ac levels and neurofibrillary tangle burden. Read counts from ChIP-seq were used as outcome and models were adjusted for library size, batch, post mortem interval, cross correlation, age and gender. Mean ChIP-seq read counts and standard deviation (SD) are shown for each locus evaluated, along with regression coefficient (β) and the unadjusted p-value. Following adjustment for multiple hypothesis testing (FDR<0.05), 3 loci remained significant (boldface).

SUPPLEMENTAL EXPERIMENTAL PROCEDURES

Human Autopsy Cohort

Religious Orders Study (ROS) and Rush Memory and Aging Project (MAP) participants were free of known dementia at enrollment, agreed to annual clinical evaluations, and signed an informed consent and Anatomic Gift Act donating their brains at death (Bennett et al., 2012a, 2012b). The ROS and MAP studies were approved by the Institutional Review Board at Rush University. Modified Bielschowsky silver stain was used to visualize neuritic plaques, diffuse plaques, and neurofibrillary tangles in tissue sections from the midfrontal, middle temporal, inferior parietal, and entorhinal cortices and the hippocampal CA1 sector (Bennett et al., 2006). As in prior work (Bennett et al., 2009), a quantitative composite score for neurofibrillary tangle or neuritic amyloid plaque pathologic burden was created by dividing the raw counts in each region by the population standard deviation of the region-specific counts and then averaging the scaled counts over the 5 brain regions to create standardized summary measures. Neuropathologic diagnosis of AD was made based on intermediate or high likelihood of AD by criteria from the National Institute on Aging and the Reagan Institute Working Group on Diagnostic Criteria for the Neuropathological Assessment of Alzheimer's Disease (National Institute on Aging, 1997). Level of cognition was based on 17 cognitive tests performed at annual clinical evaluations—the last evaluation within the year proximate to death was used. As in prior studies (Bennett et al., 2006), test results were converted to z-scores, using the mean and SD from the baseline evaluation of all participants, and averaged to yield a global summary measure of cognitive performance proximate to death. RNA-seq data was generated using postmortem brain tissue (dorsolateral prefrontal cortex) from ROS/MAP subjects, as previously described (De Jager et al., 2014). The RNA was extracted from the tissue after the quality control evaluation based on RIN (Schroeder et al., 2006) or RNA were extracted first, and then RIN was used to filter out low-quality samples. The library was sequenced using Illumina Hi-Seq with 101 bp reads and 4-plex pooling. 636 subjects had complete data including RNA-seq transcriptome profiles, neurofibrillary tangle burden outcome trait, and the pertinent covariates (Table S1). H3K9Ac chromatin-immunoprecipitation sequencing (ChIP-Seq) was also available from 675 ROSMAP brains (Klein et al., 2018; Ng et al., 2017).

Estimation and Analysis of TE Expression from Human Brain Transcriptomes

ROSMAP RNA-seq data were converted from binary sequence alignment map (BAM) format to FASTQ using Picard (https://broadinstitute.github.io/picard/). The consensus cDNA sequence library of human TEs was downloaded using the CENSOR webserver tool in Repbase (version 22.05, http://www.girinst.org/repbase/update/browse.php). As detailed in Figure S1A, our custom TE reference library included 687 retrotransposons for initial quantification from RNA-seq data, after excluding both simple repeats and multicopy genes, DNA transposons (which replicate without an RNA intermediate), and those elements lacking sufficient consensus sequence features to allow unambiguous classification in Repbase. We next used SalmonTE (version 0.6.0) (Jeong et al., 2018), which implements the Salmon algorithm (Patro et al. 2017) for efficient, alignment-free quantification of RNA-seq data to generate normalized TE counts (TPM, Transcripts Per Kilobase Million) for each ROSMAP brain transcriptome. In our prior published work (Jeong et al., 2018), the SalmonTE estimation algorithm yields comparable estimates to both qPCR and TETranscripts (Jin et al., 2015), a published tool for detection of TE expression from RNA-seq data. In selected cases, estimates of TE expression (HERV-Fc1, L1MB4, and AluYc5) in human brains were independently confirmed by PCR (Figure S1B), using the following primers:

HERV-Fc1	5'-TTGTCAGAGGCGAGGCAAAT-3'
	5'-TTAACGGCAAGAGAGGCTGG-3'
L1MB4	5'-AGACCACCTAAAGCCTGGGA-3'
	5'-GGCACTCCCCTTCAACACTT-3'
AluYc5	5'-GGAGATCGAGACCACGGTGA-3'
	5'-ACTGCAAGCTCCGCCTTCCG-3'

All TPM values were adjusted for potential RNA-seq batch effects using Combat (Johnson et al., 2007). Prior to batch correction, we excluded 140 TEs with low variability (TPM variance < 0.1), and the remaining count data for 547 TEs were quantile normalized and log2-transformed. The batch-corrected, log-transformed Transcripts Per Kilobase Million (TPM) outcome variable follows a Gaussian distribution and is suitable for linear regression. As in prior studies (Bennett et al., 2009), the neurofibrillary tangle trait was square-root transformed to approximate a normal distribution. We also included pertinent covariates to adjust for age at death, postmortem interval (PMI) and

RNA integrity (RIN). Thus, using the *lm* function in R our primary analysis tested the following statistical model: Combat(log(TPM)) ~ sqrt(NFT) + age of death + PMI + RIN. In order to account for multiple-comparisons, the Benjamini-Hochberg procedure was applied (Benjamini and Hochberg, 1995); statistical significance was based on a false discovery rate (FDR) < 0.1. Complete results of the association tests for 547 TEs with tangle pathologic burden is detailed in Table S5. The top-ranked TEs were secondarily evaluated for associations with the square-root transformed neuritic amyloid plaque trait (Table 1). In order to evaluate summary associations for each TE clade, t-statistic values were extracted from our primary regression model (Table S5), and aggregated for each clade, based on Repbase classifications. A one-sample t-test (two-tailed) was then performed to evaluate for a non-zero mean t-statistic value within each clade (Figure 1 & Table S2). Secondary analyses additionally examined associations between each TE clade and either consensus AD pathologic diagnosis (Bennett et al., 2006; National Institute on Aging, 1997) or global cognitive performance (Wilson et al., 2015), using logistic or linear regression, respectively. All analyses were adjusted for age at death, PMI and RIN, and the analysis for cognitive performance was additionally adjusted for sex and years of education.

For analysis of neurofibrillary tangle-associated chromatin changes, *HERV-Fc1* genomic loci were determined using the Dfam database and webserver (Hubley et al., 2015), based on the trusted and non-redundant filtering parameters. A 3kb genomic region was defined centered on each *HERV-Fc1* site, and we extracted reads mapped to these regions from the ChIP-seq data. Non-uniquely mapped reads and duplicated reads were removed. Regions with a mean of less than 10 reads across all samples were excluded. A log-linear regression model was implemented to evaluate associations between neurofibrillary tangle pathologic burden and level of H3K9Ac, and adjusting for library size, batch, pmi, cross-correlation, age and gender.

Drosophila Stocks and Husbandry

The *UAS-Tau*^{WT} and *UAS-Tau*^{R406W} transgenic flies were previously described (Wittmann et al., 2001). Pan-neuronal expression was achieved using the GAL4-UAS system (Brand and Perrimon, 1993), in which Tau cDNAs under the control of the yeast upstream activation sequence (UAS) are responsive to the *GAL4* transcriptional activator, directed to the nervous system via *ELAV* regulatory elements (*ELAV-GAL4*). Experimental animals consisted of the following genotypes: *ELAV-GAL4/+;UAS-Tau*^{WT}*/+* and *ELAV-GAL4/+;UAS-Tau*^{R406W}*/+*. *ELAV-GAL4/+* was used as a control. All flies were raised on standard *Drosophila* media at 25°C, and aged to 1-, 10-, or 20-days.

Analysis of TE Expression in *Drosophila* Heads

Approximately 100 adult flies, equally divided between males and females, were collected for each genotype at the specified ages. Frozen fly heads were homogenized in Trizol (Invitrogen) and treated with DNaseI (Promega). Total RNA was extracted using the RNeasy Micro Kit (Qiagen). Following reverse transcription using the SuperScript III First-Strand Synthesis System (Invitrogen), quantitative reverse transcription PCR was performed using iQ SYBR Green Supermix (Biorad) in a CFX96 Touch Real-Time PCR Detection System (Biorad) with standard cycling parameters. Each reaction was performed in triplicate. *RpL32* was used as endogenous control for normalization of each transposon to calculate ΔC_T values. We used the following PCR primers:

accord (Chung et al., 2007)	5’-CGTGAGTTACGGGTGCCTCCG-3’
	5’-AGTTACCATGCCCAGCATTAAC-3’
copia (Kalmykova et al., 2005)	5’-GCATGAGAGGTTTGGCCATATAAGC-3’
	5’-GGCCCACAGACATCTGAGTGTACTACA-3’
gate (Klenov et al., 2011)	5’-CCTTTGGGAAGCAGGCGTAAA-3’
	5’-TCTTCAGACATAGGAGAGAGCGGC-3’
het-a	5’-CGGCGAACGAGTTCTGGAAT-3’
	5’- AGTTGTGTACTTGGGCTGGG-3’
r2	5’-TCCATTCTGCCACCCTGAAC-3’
	5’-GGAAAACCCGCTCAGGTACA-3’
rt1b	5’-TGGCCAACAGAGTGGATGTC-3’
	5’-ACTTCGGTGAGCAGGATGTG-3’
qbert	5’-TATACGGTCGCCTGTGAAGC-3’
	5’-GGCAGTGGTTGTGGGGATTT-3’
tabor	5’-GACTCTCTTAGACAGCGGCG-3’
	5’-ACGACTCCTTTACGACTGCC-3’
blood	5’-GAGGGGAGGTGTAGTATGTGC-3’

	5’-GCGTGGGGATGCTGACTTAG-3’
ivk	5’-TCAAGCCACCAACGAACGTA-3’
	5’-ATTGACTGCAGGCGTTGAGA-3’
copia2	5’-CGGATTTCGTGTGTCGTTCG-3’
	5’-CCGAATCTCTGGTGCCACTT-3’
gypsy (Liu et al., 2011)	5’-GTTCATACCCTTGGTAGTAGC-3’
	5’-CAACTTACGCATATGTGAGT-3’
RpL32 (Luce-Fedrow et al., 2008)	5’-ATCGGTTACGGATCGAACAA-3’
	5’-GACAATCTCCTTGCGCTTCT-3’

One-way ANOVA was performed to detect differences between group mean expression values, considering each TE and timepoint separately. Subsetted t-tests (two-tailed) were subsequently performed for post-hoc comparisons of each Tau genotype with control animals. For *copia*, a two-way ANOVA was secondarily used to differentiate genotype and age effects. Error bars in all analyses represent the standard error of the mean (SEM). In order to control for potential confounding due to genetic background, follow-up experiments were also performed to detect *gypsy, copia,* and *het-a* expression after the *UAS-Tau*^{WT} and *UAS-Tau*^{R406W} strains were back-crossed to *w*^{1118} flies for 5 generations (Figure S2C). For estimation of TE genomic copy number (Figure S2D), 20 frozen adult fly heads, equally divided between males and females, were collected for each genotype in triplicate. Genomic DNA was extracted using the PureLink Genomic DNA Mini Kit (ThermoFisher). Quantitative reverse transcription PCR was performed using the protocol and the primer sets for *copia*, *gypsy*, and *het-a* described above. *RpL32* was again used as an internal control.

For RNA-seq, total RNA was extracted from triplicate samples consisting of 100 frozen fly heads each from either 20-day-old *ELAV>Tau*^{WT} or controls (*ELAV-GAL4/+*), and sequencing was performed at the Broad Institute using Illumina HiSeq with 96bp paired-end reads. *Drosophila* TE annotations were downloaded from Repbase (version 22.05), and TE expression signatures were determined using SalmonTE, as above (Jeong et al., 2018). Statistical analysis was performed using DESeq2, implemented within SalmonTE, and using default parameters; significance was set to FDR<0.1. Comprehensive results of the *Drosophila* RNA-seq analysis are shown in Table S6.

SUPPLEMENTAL REFERENCES

Benjamini, Y., and Hochberg, Y. (1995). Controlling the false discovery rate: a practical and powerful approach to multiple testing. J. R. Stat. Soc. *57*, 289–300.

Bennett, D.A., Schneider, J.A., Arvanitakis, Z., Kelly, J.F., Aggarwal, N.T., Shah, R.C., and Wilson, R.S. (2006). Neuropathology of older persons without cognitive impairment from two community-based studies. Neurology *66*, 1837–1844.

Bennett, D.A., De Jager, P.L., Leurgans, S.E., and Schneider, J.A. (2009). Neuropathologic intermediate phenotypes enhance association to Alzheimer susceptibility alleles. Neurology *72*, 1495–1503.

Bennett, D.A., Schneider, J.A., Arvanitakis, Z., and Wilson, R.S. (2012a). Overview and findings from the religious orders study. Curr. Alzheimer Res. *9*, 628–645.

Bennett, D.A., Schneider, J.A., Buchman, A.S., Barnes, L.L., Boyle, P.A., and Wilson, R.S. (2012b). Overview and findings from the rush Memory and Aging Project. Curr. Alzheimer Res. *9*, 646–663.

Brand, A.H., and Perrimon, N. (1993). Targeted gene expression as a means of altering cell fates and generating dominant phenotypes. Development *118*, 401–415.

Chung, H., Bogwitz, M.R., McCart, C., Andrianopoulos, A., Ffrench-Constant, R.H., Batterham, P., and Daborn, P.J. (2007). Cis-regulatory elements in the accord retrotransposon result in tissue-specific expression of the Drosophila melanogaster insecticide resistance gene Cyp6g1. Genetics *175*, 1071–1077.

Hubley, R., Finn, R.D., Clements, J., Eddy, S.R., Jones, T.A., Bao, W., Smit, A.F.A., and Wheeler, T.J. (2015). The Dfam database of repetitive DNA families. Nucleic Acids Res. *44*, D81–D89.

De Jager, P.L., Srivastava, G., Lunnon, K., Burgess, J., Schalkwyk, L.C., Yu, L., Eaton, M.L., Keenan, B.T., Ernst, J., McCabe, C., et al. (2014). Alzheimer's disease: Early alterations in brain DNA methylation at ANK1, BIN1, RHBDF2 and other loci. Nat. Neurosci. *17*, 1156–1163.

Jeong, H., Yalamanchili, H.K., Guo, C., Shulman, J.M., and Liu, Z. (2018). An ultra-fast and scalable quantification pipeline for transposable elements from next generation sequencing data. Pac. Symp. Biocomput. *23*, 168–179.

Jin, Y., Tam, O.H., Paniagua, E., and Hammell, M. (2015). TEtranscripts: A package for including transposable elements in differential expression analysis of RNA-seq datasets. Bioinformatics *31*, 3593–3599.

Johnson, W., Li, C., and Rabinovic, A. (2007). Adjusting batch effects in microarray expression data using empirical Bayes methods. Biostatistics *8*, 118–127.

Kalmykova, A.I., Klenov, M.S., and Gvozdev, V.A. (2005). Argonaute protein PIWI controls mobilization of retrotransposons in the Drosophila male germline. Nucleic Acids Res. *33*, 2052–2059.

Klein, H.-U., McCabe, C., Gjoneska, E., Sullivan, S.E., Kaskow, B.J., Tang, A., Smith, R. V, Xu, J., Pfenning, A.R., Bernstein, B.E., et al. (2018). Epigenome-wide study uncovers tau pathology-driven changes of chromatin organization in the aging human brain. bioRxiv 273789. doi: https://doi.org/10.1101/273789

Klenov, M.S., Sokolova, O.A., Yakushev, E.Y., Stolyarenko, A.D., Mikhaleva, E.A., Lavrov, S.A., and Gvozdev, V.A. (2011). Separation of stem cell maintenance and transposon silencing functions of Piwi protein. Proc. Natl. Acad. Sci. *108*, 18760–18765.

Liu, L., Qi, H., Wang, J., and Lin, H. (2011). PAPI, a novel TUDOR-domain protein, complexes with AGO3, ME31B and TRAL in the nuage to silence transposition. Development *138*, 1863–1873.

Luce-Fedrow, A., Von Ohlen, T., Boyle, D., Ganta, R.R., and Chapes, S.K. (2008). Use of Drosophila S2 cells as a

model for studying Ehrlichia chaffeensis infections. Appl. Environ. Microbiol. *74*, 1886–1891.

National Institute on Aging, Reagan Institute Working Group on Diagnostic Criteria for the Neuropathological Assessment of Alzheimer's Disease. (1997). Consensus Recommendations For the Postmortem Diagnosis Of Alzheimers-Disease. Neurobiol. Aging *18*, S 1-S 2.

Ng, B., White, C.C., Klein, H.U., Sieberts, S.K., McCabe, C., Patrick, E., Xu, J., Yu, L., Gaiteri, C., Bennett, D.A., et al. (2017). An xQTL map integrates the genetic architecture of the human brain's transcriptome and epigenome. Nat. Neurosci. *20*, 1418.

Patro, R., Duggal, G., Love, M.I., Irizarry, R.A., and Kingsford, C. (2017). Salmon provides fast and bias-aware quantification of transcript expression. Nat. Methods *14*, 417–419.

Schroeder, A., Mueller, O., Stocker, S., Salowsky, R., Leiber, M., Gassmann, M., Lightfoot, S., Menzel, W., Granzow, M., and Ragg, T. (2006). The RIN: An RNA integrity number for assigning integrity values to RNA measurements. BMC Mol. Biol. *7*, 3.

Wilson, R.S., Boyle, P.A., Yu, L., Barnes, L.L., Sytsma, J., Buchman, A.S., Bennett, D.A., and Schneider, J.A. (2015). Temporal course and pathologic basis of unawareness of memory loss in dementia. Neurology *85*, 984–991.
